# An optimal distance cutoff for contact-based Protein Structure Networks using side-chain centers of mass

**DOI:** 10.1038/s41598-017-01498-6

**Published:** 2017-06-06

**Authors:** Juan Salamanca Viloria, Maria Francesca Allega, Matteo Lambrughi, Elena Papaleo

**Affiliations:** Computational Biology Laboratory, Danish Cancer Society Research Center, Strandboulevarden 49, 2100 Copenhagen, Denmark

## Abstract

Proteins are highly dynamic entities attaining a myriad of different conformations. Protein side chains change their states during dynamics, causing clashes that are propagated at distal sites. A convenient formalism to analyze protein dynamics is based on network theory using Protein Structure Networks (PSNs). Despite their broad applicability, few efforts have been devoted to benchmarking PSN methods and to provide the community with best practices. In many applications, it is convenient to use the centers of mass of the side chains as nodes. It becomes thus critical to evaluate the minimal distance cutoff between the centers of mass which will provide stable network properties. Moreover, when the PSN is derived from a structural ensemble collected by molecular dynamics (MD), the impact of the MD force field has to be evaluated. We selected a dataset of proteins with different fold and size and assessed the two fundamental properties of the PSN, i.e. hubs and connected components. We identified an optimal cutoff of 5 Å that is robust to changes in the force field and the proteins. Our study builds solid foundations for the harmonization and standardization of the PSN approach.

## Introduction

Proteins are complex and highly dynamic entities attaining a myriad of different conformations in solution^1–5^ that are often related to the protein function. Indeed, they can resemble bound states to a biological partner^6–10^, active states of enzymes^11–14^, or conformations that are stabilized by a post-translational modification (PTM)^6, 11^, as well as altered by a disease-related mutation^15^.

An interesting property of proteins is that a perturbation (e.g. a binding event, a mutation or a PTM) occurring at a certain site of the structure can be transmitted over long distances to another location^16–19^. This long-range communication is often related to allostery and may affect critical distal sites for protein function.

At the atom-level, the perturbation from one protein site to a distal one can be propagated by a cascade of collisional clashes between residue side chains, which undergo changes of their rotameric states during protein dynamics^19, 20^. Local rearrangements occurring in the intramolecular contacts during the protein dynamics are thus at the base of this long-range communication^19^.

A convenient formalism to unravel the complexity behind long-range structural communication in proteins is the application of network theory to protein structure, i.e. the so-called Protein Structure Networks (PSNs). In a PSN, the protein residues become the nodes of the network connected by edges which can, for example, be described as the contact strength between each pair of residues^20–30^. Networks indeed are proper tools to link the local to global perturbations occurring during protein dynamics since they are by definition mediators of communication from local to global scales^19^.

Nowadays, PSN-based strategies are very popular and used in structural biology, and a plethora of different methodologies has been proposed^25–28, 31–37^. PSN approaches are often integrated to the dynamic description of proteins that all-atom molecular dynamics (MD) simulations or other sampling methods provide^21, 31, 38–45^.

Despite their broad applicability, few efforts have been devoted so far to the benchmarking of PSN and PSN-MD methods, to define best practices in the field and to ultimately provide the community with clear rules to determine PSN optimal parameters. The definition of arbitrary cutoffs is one of the major weaknesses of contact-based networks applied to protein structure and dynamics^46, 47^. As previously shown, many options are available to select suitable distance cutoffs for the prediction of residue contacts in protein structures^47^. Alternative solutions exist, i.e. using different principles for edge and weight definition such as energies or correlated motions. Nevertheless, a contact-based approach is still valuable especially if we consider the major advances that techniques such as atomistic biomolecular simulations have achieved in the last decade^48, 49^. Indeed, MD simulations have now reached high accuracy in describing conformational changes even at the side-chain levels and occurring on different time scales, as attested by the agreement with experimental observables^4, 50–53^.

In many PSN-MD applications, it is convenient to use the centers of mass of residue side chains as PSN nodes, the distance between the centers of mass for edge definition and their occurrence as weight^20, 31, 41, 54, 55^. It becomes, thus, critical to evaluate the minimal distance cutoff between the centers of mass of two residues to include an edge in the PSN. Moreover, when the PSN is derived from a structural ensemble collected by MD simulations and not from experimental structures, it is mandatory to evaluate the impact of the physical model (i.e. force field) on the PSN parameters.

We selected a dataset of proteins with different architecture and size and assessed the distribution of the two fundamental properties of a PSN, i.e. the hubs and the connected components. We also evaluated the influence of the force field selection on the PSN parameters, and we propose an optimal distance cutoff for PSN based on distances between the centers of mass of protein residues. The cutoff here identified is robust independently on the protein size, fold, and the MD force field employed. Our study builds strong foundations toward the harmonization and standardization of PSN strategies and a framework to apply also more generally to the choice of parameters for other PSN-based approaches.

## Results and Discussion

### Selected protein structures for PSN-MD analyses

**Figure 1:**
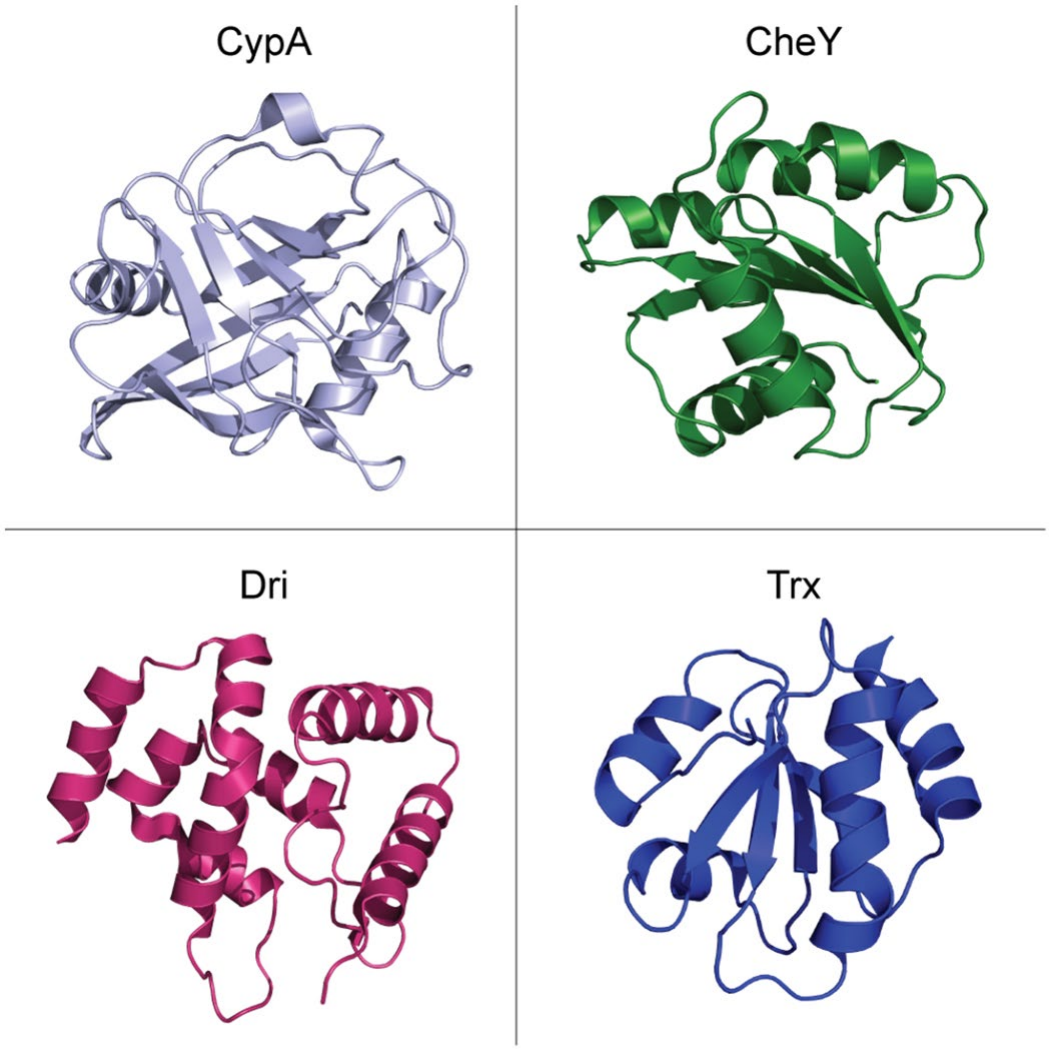
3D of the selected proteins for molecular simulations. The 3D structures of the Cyclophilin A (CypA) from *H.sapiens* (PDB entry 3K0N), Chemotaxis protein (CheY) from *E.coli* (PDB entry 3CHY), the DNA-binding domain of the Dead ringer protein (Dri) from *D.melanogaster* (PDB entry 1C20) and the Thioredoxin (Trx) from *B.acidocaldarius* (PDB entry 1QUW) are shown as light blue, green, magenta and blue cartoon, respectively.

We selected four different three-dimensional (3D) structures of monomeric proteins of various size and fold (Fig. 1) and four different force fields (Table S1). In particular, we chose state-of-the-art physical models from each of the most used force-field families for MD simulations of proteins, i.e. CHARMM (CHARMM22*^56^ and CHARMM36^57^), AMBER (Amber99SB*-ILDN^58, 59^) and GROMOS (GROMOS54a7^60^). We carried out the MD simulations in explicit solvent for one μs so that they could reflect the MD sampling that is employed for PSN-MD studies^40, 54^. For each MD ensemble, PSN based on distances between the side-chain centers of mass have been calculated as detailed in the Materials and Methods.

### A distance cutoff of 5 Å allows a robust description of PSN properties independently on the protein and the MD force field employed

The choice of the distance cutoff is essential for the PSN definition. Indeed, the distance cutoff is used to discriminate which contact between two side chains has to be included or not as a link of the network, ultimately affecting the network topology. When the distance is calculated between the centers of mass of the residue side chains, the choice of the cutoff becomes even more critical. Indeed, we cannot arbitrarily assume that the distances commonly used in structural biology to define an interaction between two amino acids - such as 4 or 4.5 Å - are valid. The issue becomes even more cogent when a PSN is derived by an MD ensemble where each force field relies on different atomic masses.

**Figure 2:**
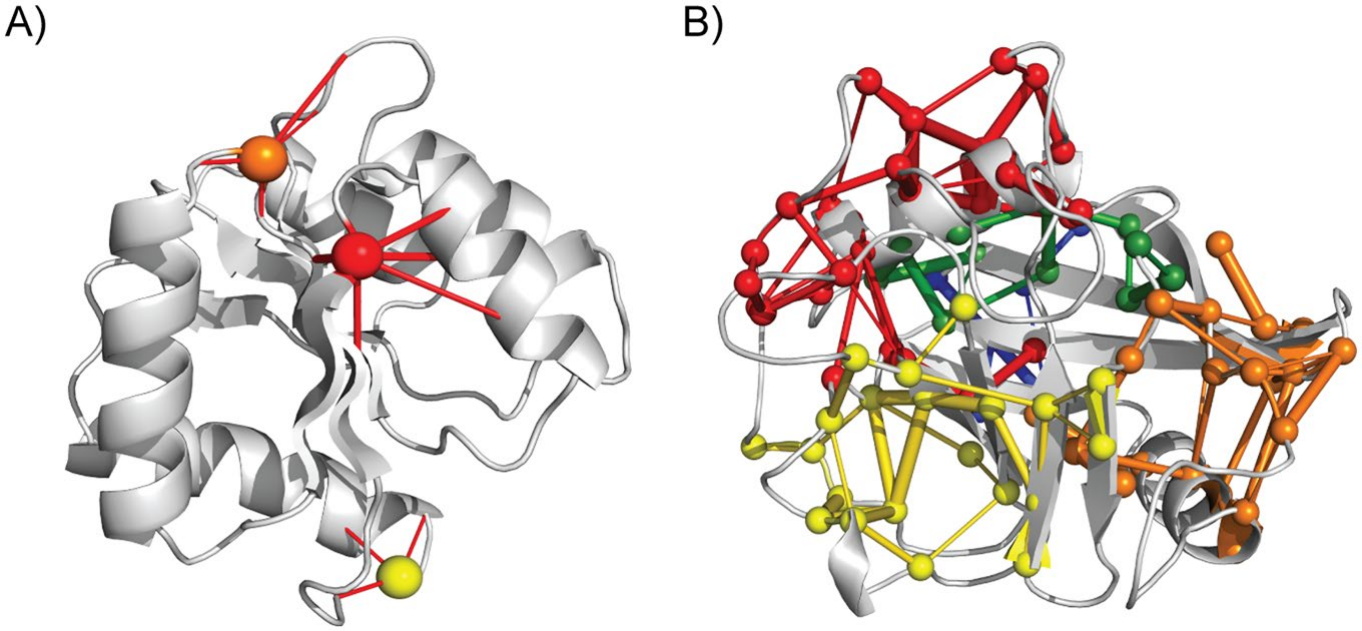
Schematic representation of hubs and connected components. Hubs are nodes that have a degree higher than the average degree of the nodes of a network. In PSN, we consider as hubs only those nodes having a number of edges greater than or equal to three. Hubs with a degree of three, four and five are shown in yellow, orange and red, respectively (**A**). The connected components are clusters of linked nodes with no edges in common with nodes that belong to the other clusters of the PSN. As an example, five connected components are shown (**B**).

The two most important properties of a PSN, which ultimately dictate how distant regions of the PSN are linked are the so-called hubs and connected components (also known as clusters of nodes) (Fig. 2).

Hubs are nodes that have a high degree of connectivity in a network. The highest degree of residue hubs is limited by steric constraints and it could vary from three to ten in PSN^27^. Protein structures are known to be made up of a significant number of strongly and weakly interacting residue hubs that stabilize the tertiary structure of the protein and provide resilience against random mutations^19, 27^.

A robust PSN should feature a certain amount of hub residues that have at least a node degree of three (i.e. connected with three or more other nodes by an edge in the PSN) and it should be composed of multiple connected components which are not too fragmented. Cluster fragmentation is particularly critical in the PSN definition. Other colleagues and we showed that central parameters that influence the size of the connected components are the *p*_*crit*_^31, 42^ or *I*_*crit*_^40, 61, 62^, depending on the methods used for PSN construction. Indeed, edges that have extremely low weights would increase the noise and connect all the clusters into a single one. Conversely, if only high weights are retained, only sparsely populated and highly fragmented clusters will be observed with a minimal number of communication paths between distal regions.

In a PSN approach based on side-chain-side-chain contacts, the distance cutoffs used can affect the network in a similar way. Indeed, if a distance that is too short and restrictive is chosen, the network will appear as very fragmented with small separated clusters and few or virtually no hubs. If the distance is too long, each residue of the network will be connected, resulting in a single cluster that embraces the entire network. It is thus critical to find an optimal distance cutoff.

Moreover, since the PSN-MD approaches, as the one here employed, generally rely on extracting an average and static PSN from an MD trajectory, it becomes fundamental to assess the convergence of hubs and connected components over the simulation time.

**Figure 3:**
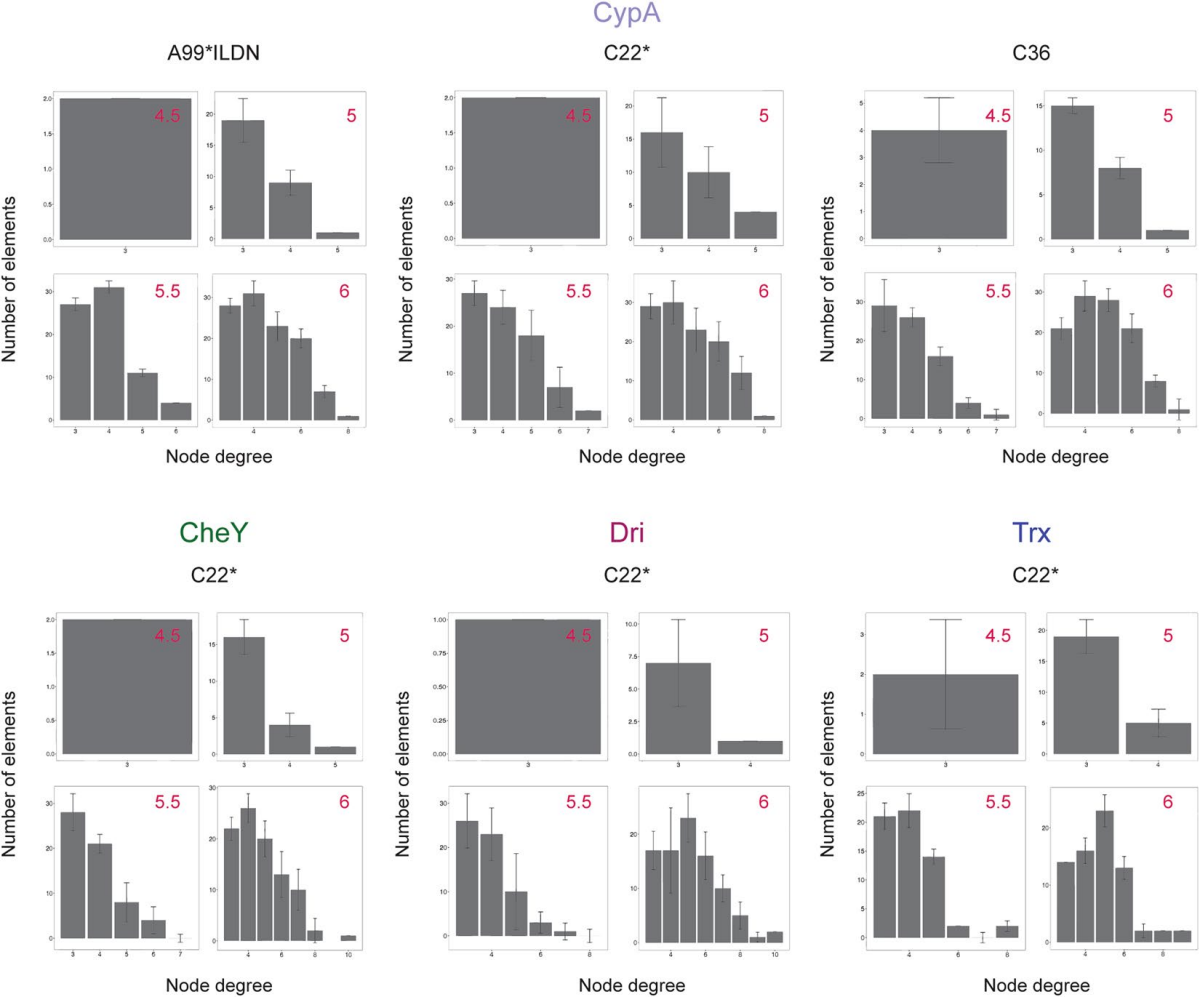
Hub distribution at different distance cutoffs used for the PSN-MD analyses. We evaluated the changes in the number of hubs and their node degree as a function of different distance cutoffs in the PSN derived from the entire MD trajectory (histogram values) and the associated standard deviations (error bars) calculated from the average PSNs obtained from the Jackknife resampled trajectories (see Materials and Methods). We noticed that hubs are virtually absent at distance cutoffs lower than 5 Å.

**Figure 4:**
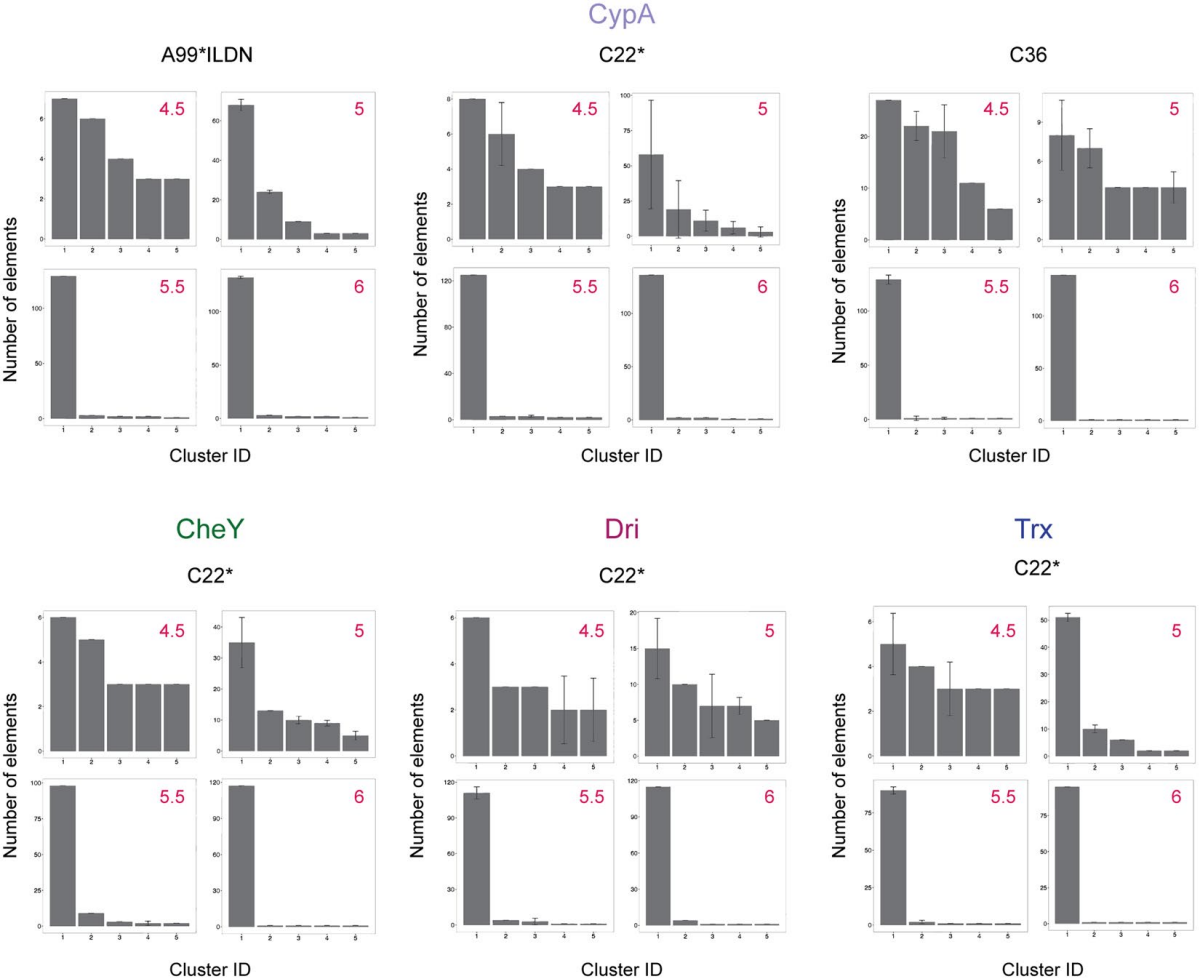
Connected component distribution at different distance cutoffs used for the PSN-MD analyses. We evaluated changes in the population of the connected components (i.e. clusters) as a function of different distance cutoffs in the PSN derived from the entire MD trajectory (histogram values) and the associated standard deviations (error bars) calculated from the average PSNs obtained from the Jackknife resampled trajectories (see Materials and Methods). We reported in the plot only the first five most populated clusters for sake of clarity. We observed that at distance cutoffs higher than 5 Å, most of the nodes of the PSN were located in the same cluster (cluster ID 1). This result suggests that 5 Å is an optimal distance cutoff to predict residue contacts in PSN.

We thus here evaluated: (i) the convergence of hubs and connected components in PSN derived by MD simulations using a Jackknife approach (see Materials and Methods) and (ii) the distribution of hubs and connected components at different distance cutoffs (Figs 3 and 4, Fig. S1). (iii) In the attempt of harmonizing the PSN protocol and allowing the reproducibility of the analyses, we also implemented a Python-based pipeline (PyInKnife.py) to automatize the steps described above, which can be used free of charge (see Materials and Methods for details).

At first, we evaluated whether hubs and connected components are stable properties in the MD ensembles here collected (Figs 3 and 4, Fig. S1). With regards to the distance cutoff, we identified common trends in the hubs and connected components distribution independently from the protein under investigation and the force field employed in the simulations. Indeed, in all the cases distance cutoffs lower than 5 Å resulted in a minimal number of hubs (less than four hub residues) where the connection degree was smaller than three (Fig. 3). On the contrary, distance cutoffs higher than 5 Å showed only one large cluster accounting for most of the protein residues (Fig. 4), indicating that this value is the more appropriate cutoff to employ for a PSN-MD where the contacts are calculated as distances between the centers of mass of residue side chains.

### Localization of hubs and connected components on the 3D structure is conserved using the 5 Å distance cutoff

The 5 Å distance cutoff allows for similar general features of the PSN of the same protein described by different force fields (Figs 3 and 4 **)**. Despite this result is encouraging, we need to take into consideration that PSNs are employed to achieve residue-level details in structural biology. PSNs are used to identify the localization of the hub residues, the specific residues that belong to the same cluster or even the paths of communication between distal residues and their intermediate nodes. These are all important PSN properties that can, for example, be altered by interactions with biological partners^6, 40, 63^ or mutations^21, 40, 42, 51, 64^. It is thus not enough to observe that the PSN description is robust regarding the overall distribution of hubs and connected components. Indeed, the PSNs collected for the same protein, but using different MD force fields, with the 5 Å distance cutoff might differ in the localization of hub residues in the 3D structure or in the individual residues that belong to the same cluster without affecting the total number of hubs and connected components. The same observation holds for the localization of hubs and connected components when the entire MD trajectory is compared to the resampled MD trajectories collected from the Jackknife approach.

We thus compared the hubs and connected components at the residue-level as derived by the PSN analyses of the entire MD trajectories or of the resampled MD trajectories obtained with the Jackknife procedure (see Materials and Methods). The analyses showed a reasonable convergence of hubs and connected components also at the residue-level with only minor discrepancies among the PSN calculated from the entire MD trajectory and few of the resampled trajectories (Figs S2 and S3).

**Figure 5:**
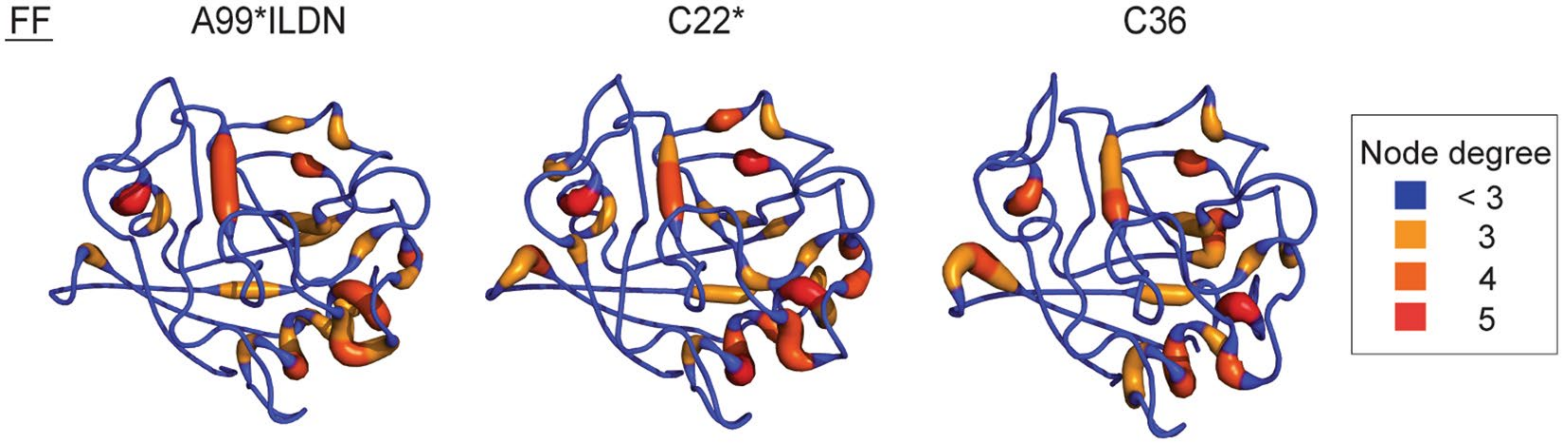
Location of the hub residues on the 3D structure of CypA. We mapped on CypA 3D structure the PSN hubs identified in CypA MD simulations with the three different force fields CHARMM22* (C22*), CHARMM36 (C36) and Amber99SB*-ILDN (A99*ILDN). The different colors and sizes represent the node degree, i.e. the number of edges for each residue.

**Figure 6:**
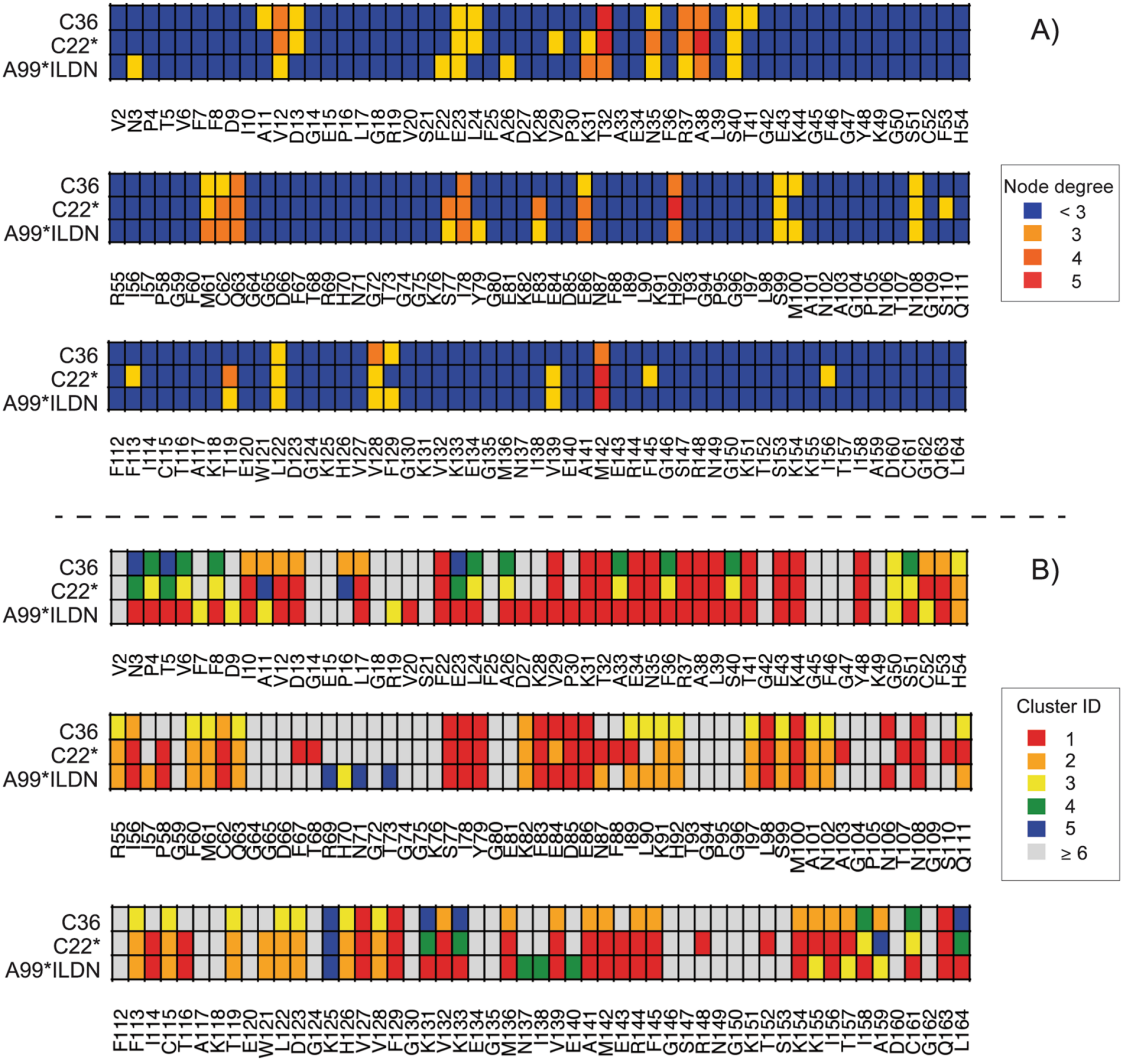
Heatmaps comparing hubs and connected components of CypA MD simulations. We used heatmaps to show the hubs (**A**) and the connected components (i.e. clusters) (**B**) identified in CypA MD simulations with the three different force fields CHARMM22* (C22*), CHARMM36 (C36) and Amber99SB*-ILDN (A99*ILDN). This representation allows to identify changes in node degree or in the cluster where a node is located at the residue-level.

Moreover, we analyzed the hub localization and their degree in the MD simulations of CypA where different force fields have been used (Figs 5 and 6A). We noticed that the localization of the hubs appears to be equally distributed on the 3D structure coming from different force fields, apart from minor changes in their node degree. Similar results were obtained for Trx using CHARMM22* and GROMOS54a7 force fields.

**Figure 7:**
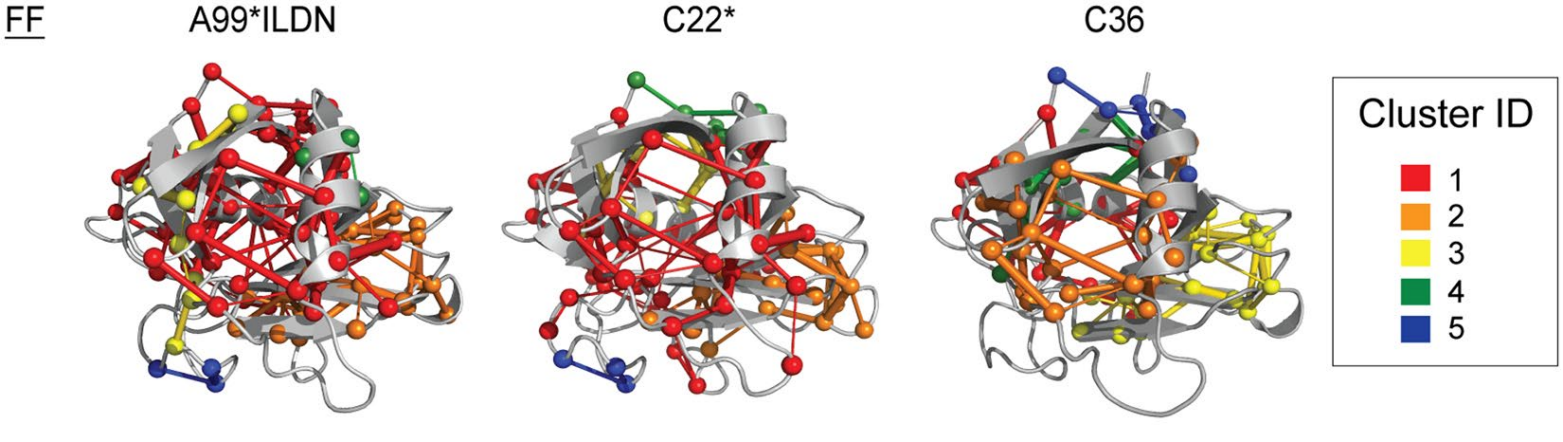
Location of the first five connected components on the 3D structure of CypA. We mapped on CypA 3D structure the PSN connected components identified in CypA MD simulations with the three different force fields CHARMM22* (C22*), CHARMM36 (C36) and Amber99SB*-ILDN (A99*ILDN). The different colors range from red to blue for the largest and smallest clusters, respectively. The nodes are represented as spheres while the edges are visualized as cylinders. The figure has been produced with the *xPyder* plugin^65^ for Pymol.

In parallel, we also mapped the first five more populated connected components onto the CypA sequence and 3D structure (Figs 6B and 7). The composition and distribution of the clusters are different only in CHARMM36 simulations. This apparent difference is only due to a splitting of the connected component number 1 in three smaller clusters, as well as to a different localization of the 5^th^ cluster (i.e. the smallest one). Only subtle differences have been observed for Amber99SB*-ILDN and CHARMM22*, suggesting a robust description of the connected components with these two force fields, as also found in a recent PSN study of a dimer^54^.

## Conclusions

In the protein world, a perturbation occurring at a certain site of the protein structure can be transmitted over long distances to another site. These structural rearrangements can be propagated by a cascade of changes in the conformational states of the residue side chains. Local changes occurring in the residue-residue contacts during the protein dynamics are thus at the base of this long-range communication. Network theory is a suitable formalism to evoke to analyze protein structures and to identify the paths of residues that can transmit the structural changes over long distances. In this context, a plethora of different approaches to define a PSN has been developed, often integrated with molecular dynamics simulations to account for the protein dynamics.

Despite the broad application of these methods, the community is missing clear rules and a solid framework to define the PSN parameters. It becomes thus critical to evaluate the minimal distance cutoff that can be used to include an edge in the PSN and that provides stable network properties, as well as the influence of the physical model used to describe the protein in the simulations.

Indeed, there are not consolidated and uniform protocols in the PSN-MD field, especially when the edges are defined according to the distance between the centers of mass of protein side chains. Moreover, most of the PSN approaches have been optimized using datasets of static experimental structures from the Protein Data Bank. A careful evaluation of the PSN parameters in an MD ensemble of structures has been poorly applied. PSN parameters that are optimal for the network analyses of experimental crystallographic structures are not necessarily suitable for the analysis of an MD ensemble, as recently pointed out^40^. Most of the publications in which a PSN was calculated using the *PyInteraph* suite of tools, for example, employ very different distance cutoffs.

We thus selected a dataset of proteins to use as model systems to assess important PSN properties as a function of different distance cutoffs and physical models. In particular, we focused on two fundamental properties of the PSN, i.e. the hubs and the connected components. We identified an optimal value for the distance cutoff (5 Å) that is robust to changes in the MD force field and applicable to proteins with different sizes or folds. Our study provides a general framework to select PSN parameters and to improve reproducibility of the results thanks to a free-of-charge Python-based pipeline, *PyInKnife*. We here built the foundations toward the harmonization and standardization of the PSN-MD approach.

## Materials and Methods

### Molecular dynamics simulations

We performed explicit solvent MD simulations using the *GROMACS* software version 4.6^66^ with different force fields and solvent models. A summary of the starting structures, protein size, force fields and solvent models used in this study is reported in Table S1. The MD simulation of Dri ARID domain has been published before^40^ and here employed for the analyses. 500-ns simulations of CypA have been published before^51^ and we here elongated them to achieve one μs of sampling. We collected the remaining simulations for the first time in this study at 300 K and 1 bar in the NVT ensemble with 150 mM of NaCl. We employed periodic boundary conditions and we set a distance equal or greater than 1.8 Å from the protein atoms and the box edges of a dodecahedral box of water molecules. Preparation steps have been carried out according to a protocol recently applied to other proteins^67^. We applied a 2-fs time step and the LINCS algorithm^68^, as well as the Particle-Mesh Ewald (PME) summation scheme^69^ to treat long-range electrostatic interactions. Van der Waals and short-range Coulomb interactions were truncated at 9 Å and conformations stored every 10 ps. We carried out productive MD simulations for one μs.

We calculated the minimal distance between each protein and its image to rule out artifacts due to periodic boundary conditions and artificial contacts between the protein and the corresponding image.

### PSN definition

We used the *PyInteraph* suite of tools^31^ to construct a PSN-MD based on side-chain contacts using all the residues except for glycines. The contacts are defined as distances between the centers of mass of side chains on the base of the atomic mass files provided by *PyInteraph*. Different distance cutoffs have been assessed in this study in the range of 4–6 Å (see below) to include a certain contact as edge of the network. Moreover, to derive a weighted network, the persistence of the contact in each MD ensemble was measured and a *p*_*crit*_ of 20% was employed to filter out meaningless interactions and to maintain the network structure, in agreement with previous applications of the same method^31, 42, 70^. We also used the *xPyder* plugin^65^ for Pymol to map on the 3D structure the PSN connected components.

### The PyInKnife pipeline

**Figure 8:**
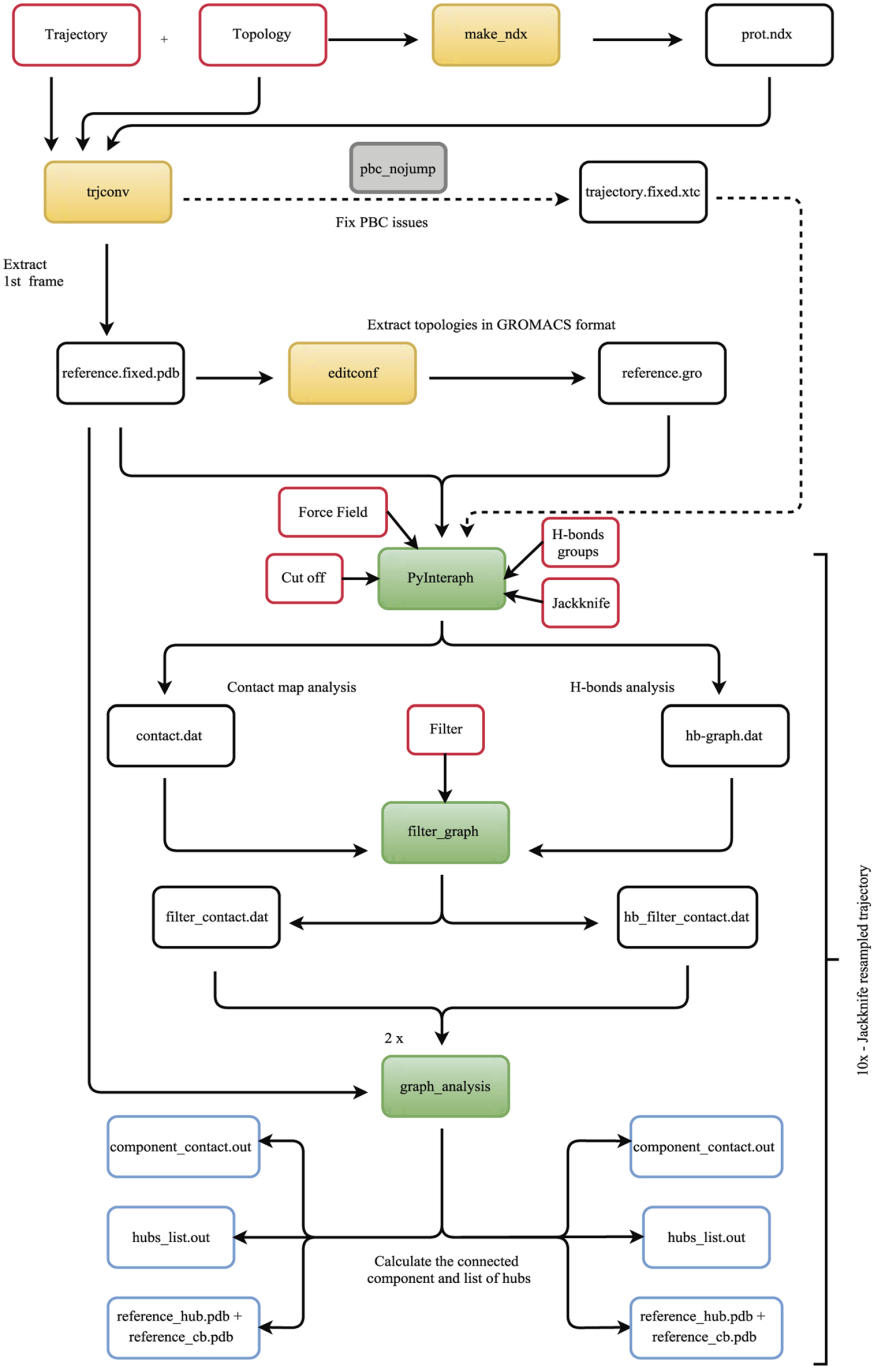
Flow chart for the *PyInKnife* pipeline for PSN analyses. The orange boxes represent the tools used from *GROMACS* software (*make_ndx*: to generate an index file consisting of the groups of interest, *trjconv*: to convert and manipulate trajectory files and *editconf*: to convert and manipulate structure files). White boxes with the red border are the main inputs of the pipeline while the boxes with blue border are the main outputs. An optional flag has been added to post-process the MD trajectories to remove artefacts related to periodic boundary conditions with *trjconv* (grey box). The green boxes represent the commands used from *PyInteraph*. The inputs required for the *PyInteraph* tool are the force field used in the MD simulation, the selected distance cutoff and the optional usage of the Jackknife resampling method. Moreover, the user can choose if to include or not the H-bonds in the PSN analysis. As an input, it is also possible to change the *p*_*crit*_ cutoff value used to remove the less informative interactions; by default this value is set to 20.

We developed a Python-based pipeline (which is available free of charge at https://github.com/ELELAB/PyInKnife) called *PyInKnife* in order to: (i) automatize the pre-processing of the trajectories for PSN analyses, (ii) sub-set the trajectories in shorter trajectory files that retain 90% of the frames (see below), (iii) run the different steps of *PyInteraph* on each trajectory subset and using different distance cutoffs, including the creation of the PSN, calculation of hubs and connected components and their distribution, and (iv) generate a final report with publication-ready plots and figures. The pipeline is illustrated in Fig. 8.

*PyInKnife* requires the pre-processing of the MD trajectory to remove artefacts due to the periodic boundary conditions and to extract a reference structure along with the topology required for the PSN calculations. The pre-processing is carried out by three different *GROMACS* tools (www.Gromacs.org): *make_ndx, trjconv* and *editconf*. These tools allow us to generate the index file, convert and manipulate the trajectories and structures, respectively.

*PyInKnife* can be also used on trajectories obtained with other simulation packages, such as *Amber, CHARMM* and *NAMD* after conversion of the MD trajectory to the *GROMACS* format (.xtc or.trr file). This can be achieved with several tools such as *WORDOM* ^71^, the *MDAnalysis* package^72^ and the *Catdcd* plugin (http://www.ks.uiuc.edu/Development/MDTools/catdcd/). The user can employ the GROMACS tool *editconf* to convert the PDB file of the starting structure, or one frame extracted from the trajectory, into the file format required by *PyInteraph* (*GROMACS*.gro file).

*PyInKnife* allows to automatize the analyses of contact-based PSN, hydrophobic interactions, and hydrogen bond networks implemented in *PyInteraph*. The user can specify from the command line the PyInteraph atomic mass databases, the distance cutoff values to be tested and other PSN parameters.

After the PSN for each MD trajectory is obtained, it is possible to calculate with *PyInKnife* the hubs and connected components for each class of interactions by using the *graph_analysis* tool of the *PyInteraph* suite.

**Figure 9:**
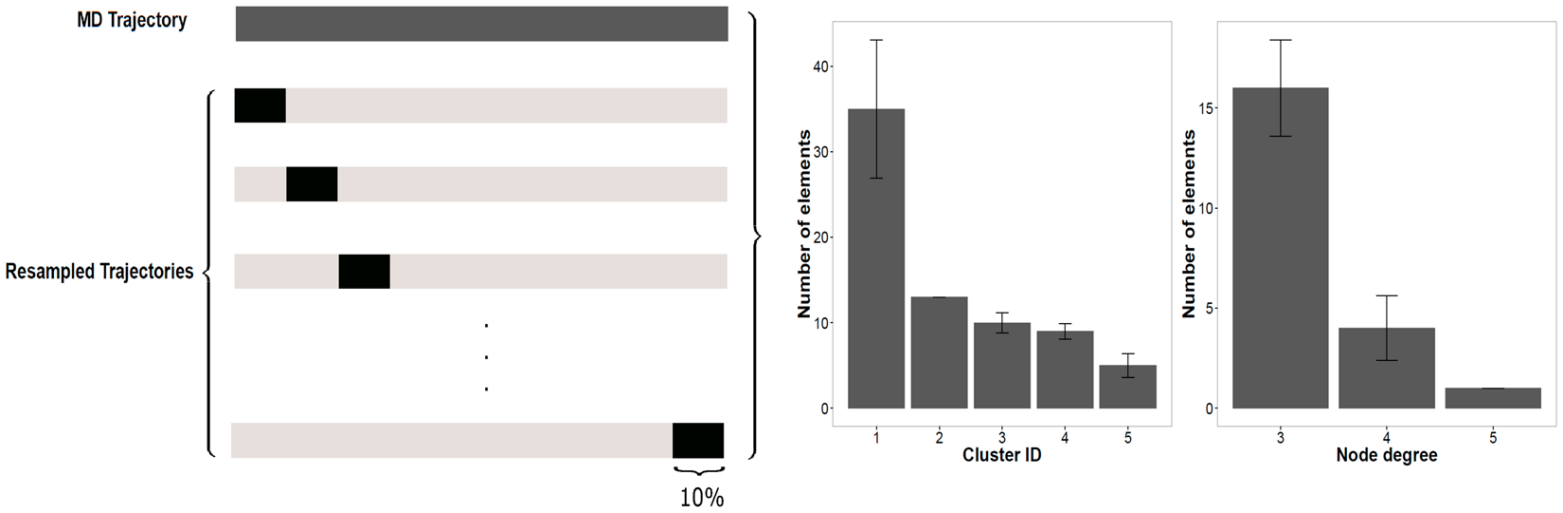
Jackknife resampling as implemented in *PyInKnife*. We show a schematic representation of the MD trajectory and the resampled trajectories used for the calculation of the convergence of PSN properties (left panel). The histograms show the two properties of the PSN analysis calculated for the whole trajectory, the connected components and the hubs (right panels). The example refers to the PSN analysis using a 5 Å distance cutoff of the CheY MD simulation with CHARMM22* force field. Error bars represent the Jackknife standard error from the resampled trajectories.

*PyInKnife* also implements a pipeline to evaluate the convergence of the two most important PSN properties, i.e. hubs and connected components in the MD trajectory. We used the Jackknife resampling method^73^ to calculate the deviation from resampled trajectories where a 10% has been discarded at regular intervals of the simulation frames. The resampled trajectories are calculated using the *GROMACS* tool *trjcat*. The procedure is illustrated in Fig. 9.

*PyInKnife* also includes R-based scripts to plot the results and produce publication-ready figures. To use the plotting R scripts, the R packages *ggplot, ggplot2* and *lattice* are required.

The Jackknife standard error is calculated as

$$SE{(\hat{x})}_{jack}={\{\frac{n-1}{n}\sum _{i=1}^{n}{({\hat{\theta }}_{(i)}-{\hat{\theta }}_{(.)})}^{2}\}}^{1/2}$$

where *n* is the number of resampled trajectories (10 as default), $\hat{\theta }$ is the estimator of the *ith* resampled trajectory and ${\hat{\theta }}_{(.)}$ is the empirical average of the estimator on the resampled trajectories

$${\hat{\theta }}_{(.)}=\frac{1}{n}\sum _{i=1}^{n}{\hat{\theta }}_{(i)}$$

## Electronic supplementary material

Supplementary Information
