# Supplementary material for: An optimal distance cutoff for contact-based Protein Structure Networks using side-chain centers of mass
Source: Sci Rep. 2017 Jun 6;7:2838. doi: 10.1038/s41598-017-01498-6 (PMC5460117; doi:10.1038/s41598-017-01498-6)
Supplement: Supplementary file 1 — Supplementary Information [file 41598_2017_1498_MOESM1_ESM.pdf]

An optimal distance cutoff for contact-based Protein Structure Networks using side-chain centers of mass.

Juan Salamanca Viloria[1], Maria Francesca Allega[1], Matteo Lambrughi[1], Elena Papaleo[1]*

[1]Computational Biology Laboratory, Danish Cancer Society Research Center, Strandboulevarden 49, 2100, Copenhagen, Denmark

*corresponding author: elenap@cancer.dk

Table S1. Summary of the MD simulations collected in this study. We predicted the resolution value for NMR structures with *Resprox*.

System	Model	PDB Entry	Force field	N aa	Method	Resolution	References (for the structure)	References (for the simulations)
CheY	Chemotaxis Protein	3CHY	CHARMM22*/Tip3p	128	X-RAY Diffraction	1.66 Å	Crystal structure of Escherichia coli CheY refined at 1.7-A resolution. (1991) Volz, K., Matsumura, P. *J.Biol.Chem*. 266: 15511-15519	
CypA	Cyclophilin A	3K0N	A99*ILDN/Tip3p<br>CHARMM22*/Tip3p<br>CHARMM36/Tip3p	165	X-RAY Diffraction	1.39 Å	Hidden alternative structures of proline isomerase essential for catalysis. (2009) Fraser, J.S., Clarkson, M.W., Degnan, S.C., Erion, R., Kern, D., Alber, T. *Nature* 462: 669-673	We here extended to one μs the 500-ns MD simulations published in: Conformational Changes and Free Energies in a Proline Isomerase. (2014) Papaleo, E., Sutto, L., Gervasio, F.L., Lindorff-Larsen, K. *J. Chem. Theory Comput.* 10:4169-74
Dri	Dead Protein Ringer DNA binding domain	1C20	CHARMM22*/Tip3p	128	SOLUTION NMR	3.00 Å	Solution structure of the DNA binding domain from Dead ringer, a sequence-specific AT-rich interaction domain (ARID). (1999) Iwahara, J., Clubb, R.T. EMBO J. 18: 6084-6094	Communication routes in ARID domains between distal residues in helix 5 and the DNA-binding loops. (2014) Invernizzi, G., Tiberti, M., Lambrughi, M., Lindorff-Larsen, K., Papaleo, E. *PLoS Comput Biol.* 10(9): e1003744.
Trx	Thioredoxin	1QUW	CHARMM22*/Tip3p<br>GROMOS54/Spc216	105	SOLUTION NMR	1.68 Å	NMR solution structure of a novel thioredoxin from Bacillus acidocaldarius possible determinants of protein stability. (2000) Nicastro, G., De Chiara, C., Pedone, E., Tato, M., Rossi, M., Bartolucci, S. *Eur.J.Biochem.* 267: 403-413	

Figure S1. Distribution of hub and connected components for Trx MD simulations with GROMOS54a7. Trx was also simulated using the GROMOS54a7 (GROMOS54) force field. The PSN analysis showed a distribution of the hubs residues and of the connected components in line with the results obtained with the other force fields (see Main Text **Fig 2-3**). The main chain root mean square deviation of the simulation over time is also shown.

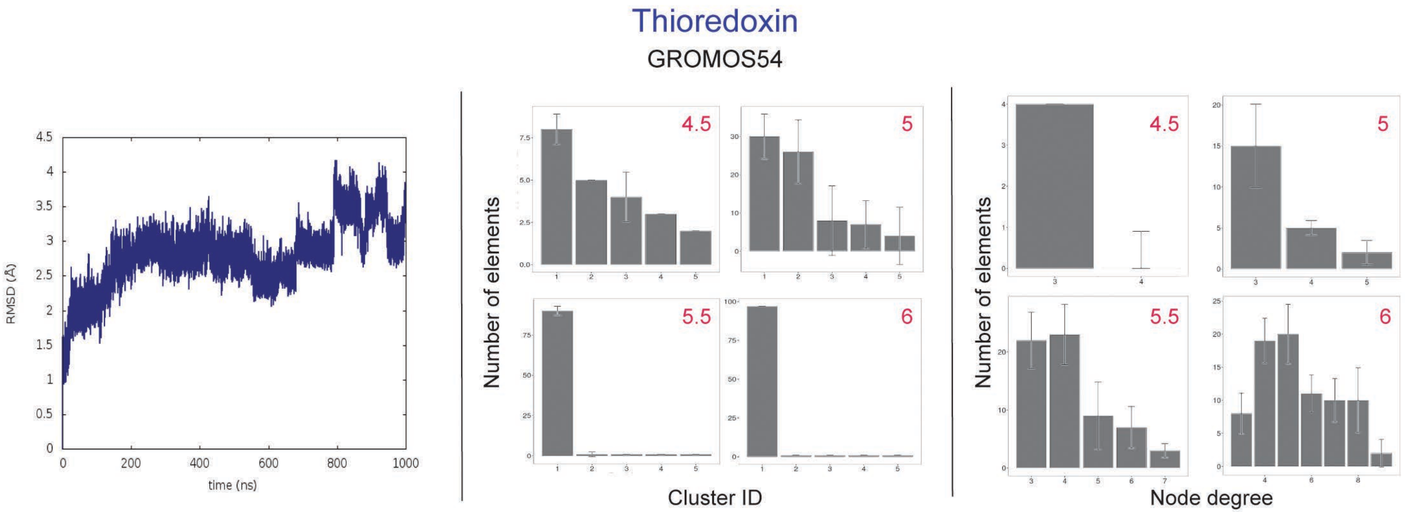

Figure S2. Heatmaps of hubs from all the MD simulations. The node degree as derived by each simulation using the Jackknife procedure are shown in the heatmaps.

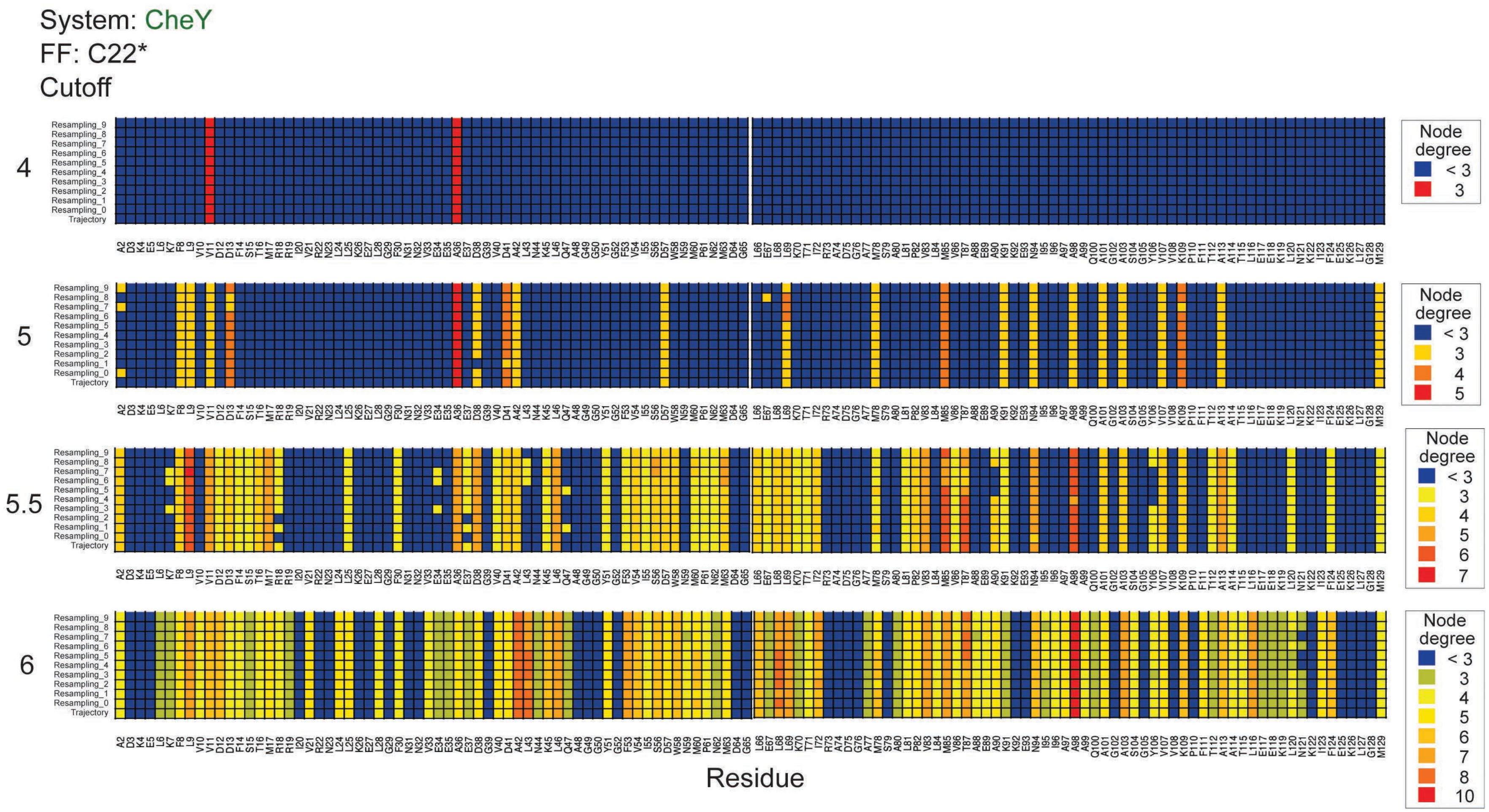

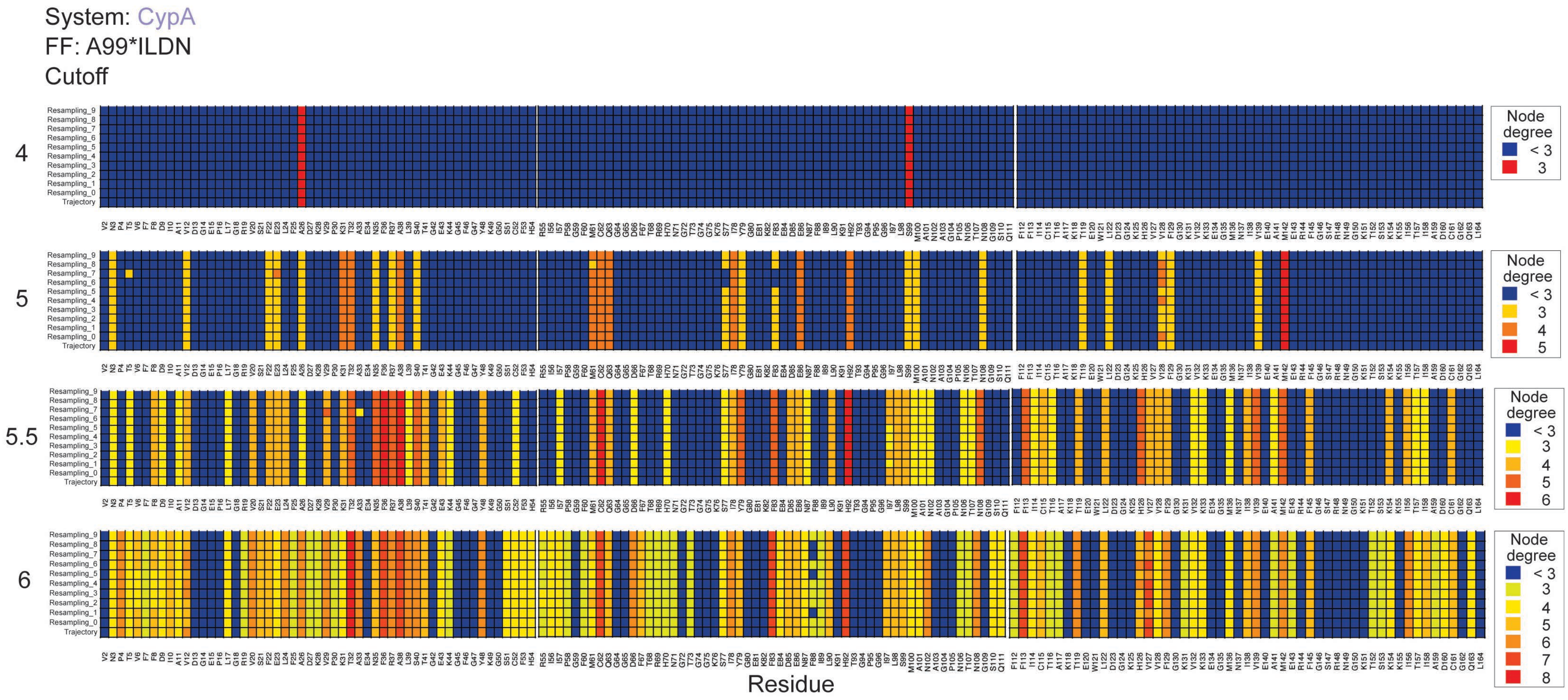
System: CypA
FF: A99*ILDN
Cutoff
4
5
5.5
6
Resampling_9
Resampling_8
Resampling_7
Resampling_6
Resampling_5
Resampling_4
Resampling_3
Resampling_2
Resampling_1
Resampling_0
Trajectory
Node degree
< 3
3
4
5
6
7
8
Residue

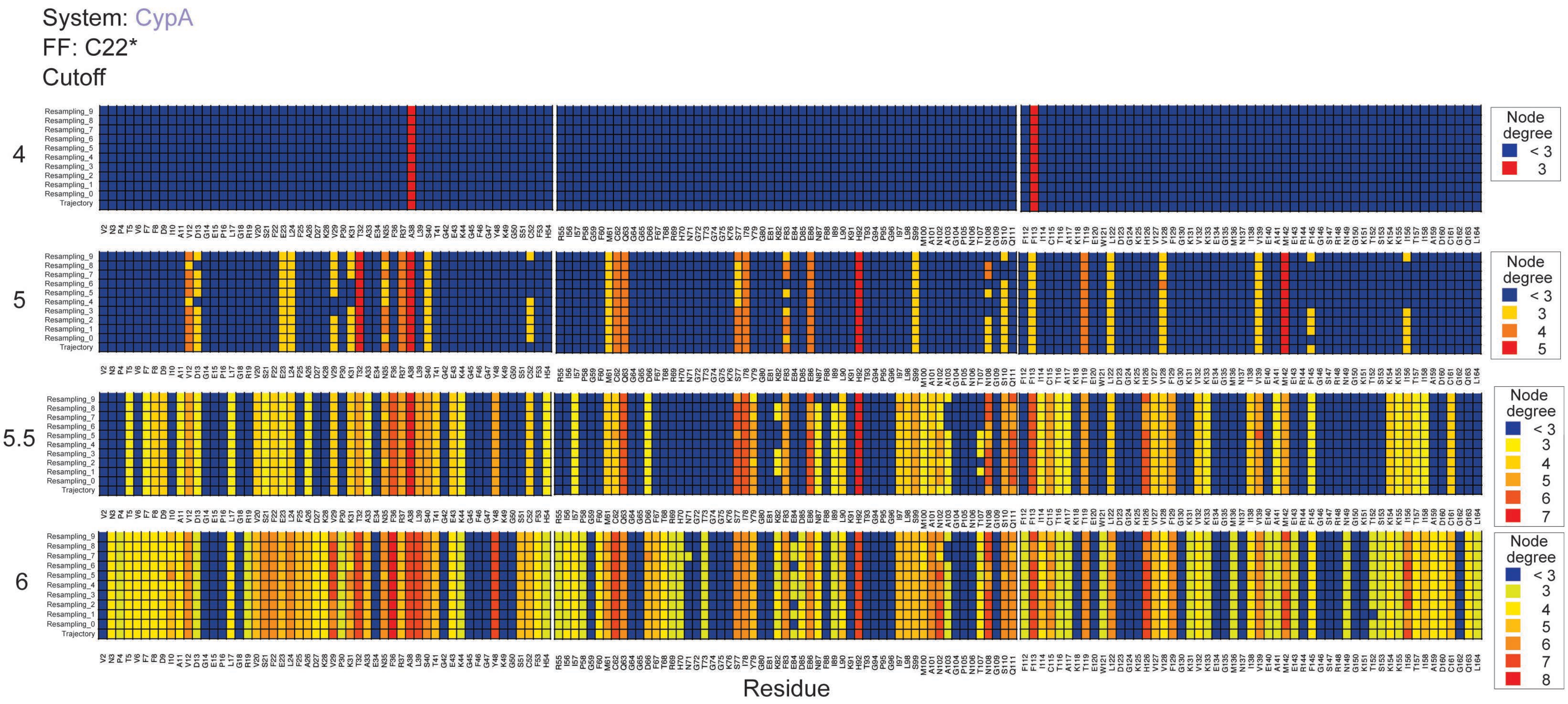
System: CypA
FF: C22*
Cutoff
4
5
5.5
6
Resampling_9
Resampling_8
Resampling_7
Resampling_6
Resampling_5
Resampling_4
Resampling_3
Resampling_2
Resampling_1
Resampling_0
Trajectory
Node degree
< 3
3
4
5
6
7
8
Residue

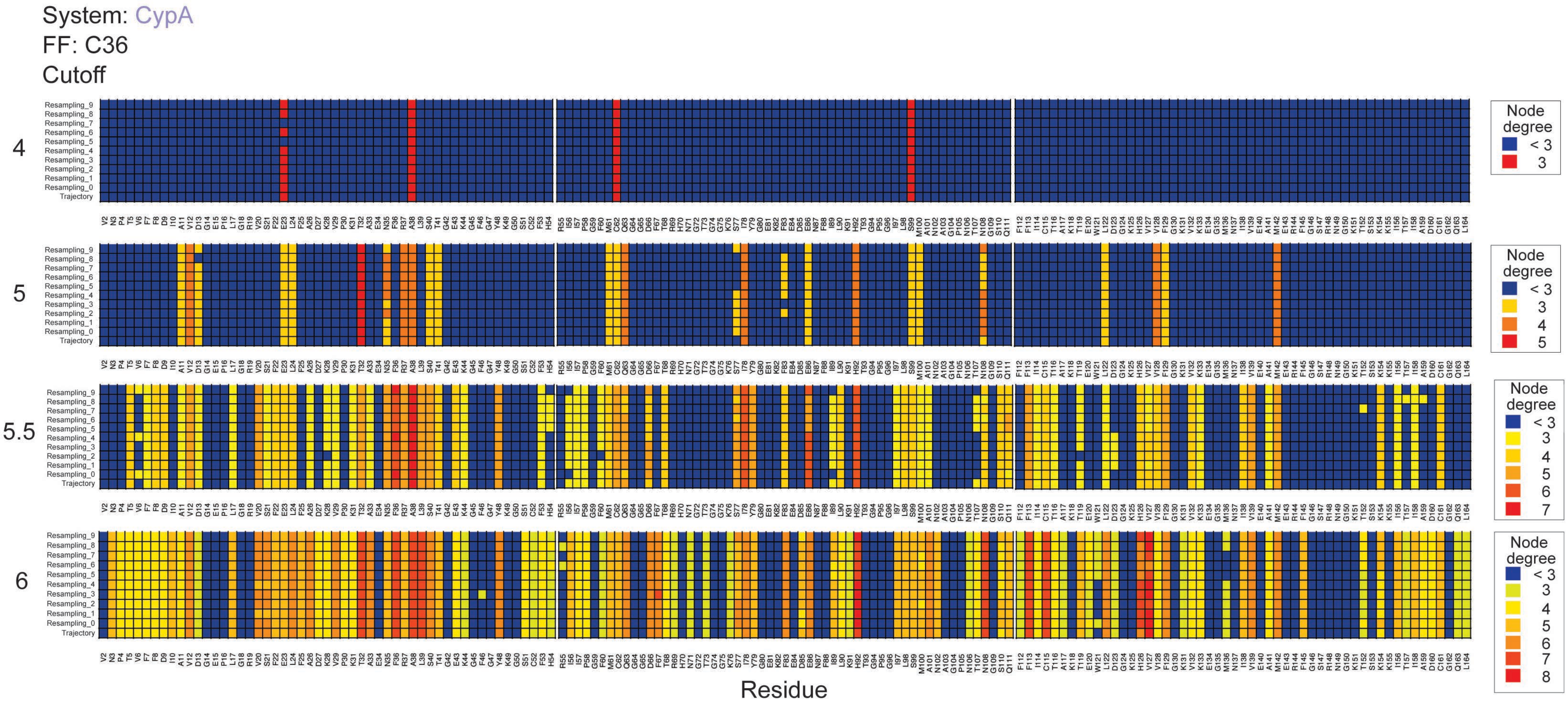
System: CypA
FF: C36
Cutoff
4
5
5.5
6
Resampling_9
Resampling_8
Resampling_7
Resampling_6
Resampling_5
Resampling_4
Resampling_3
Resampling_2
Resampling_1
Resampling_0
Trajectory
Node degree
< 3
3
4
5
6
7
8
Residue

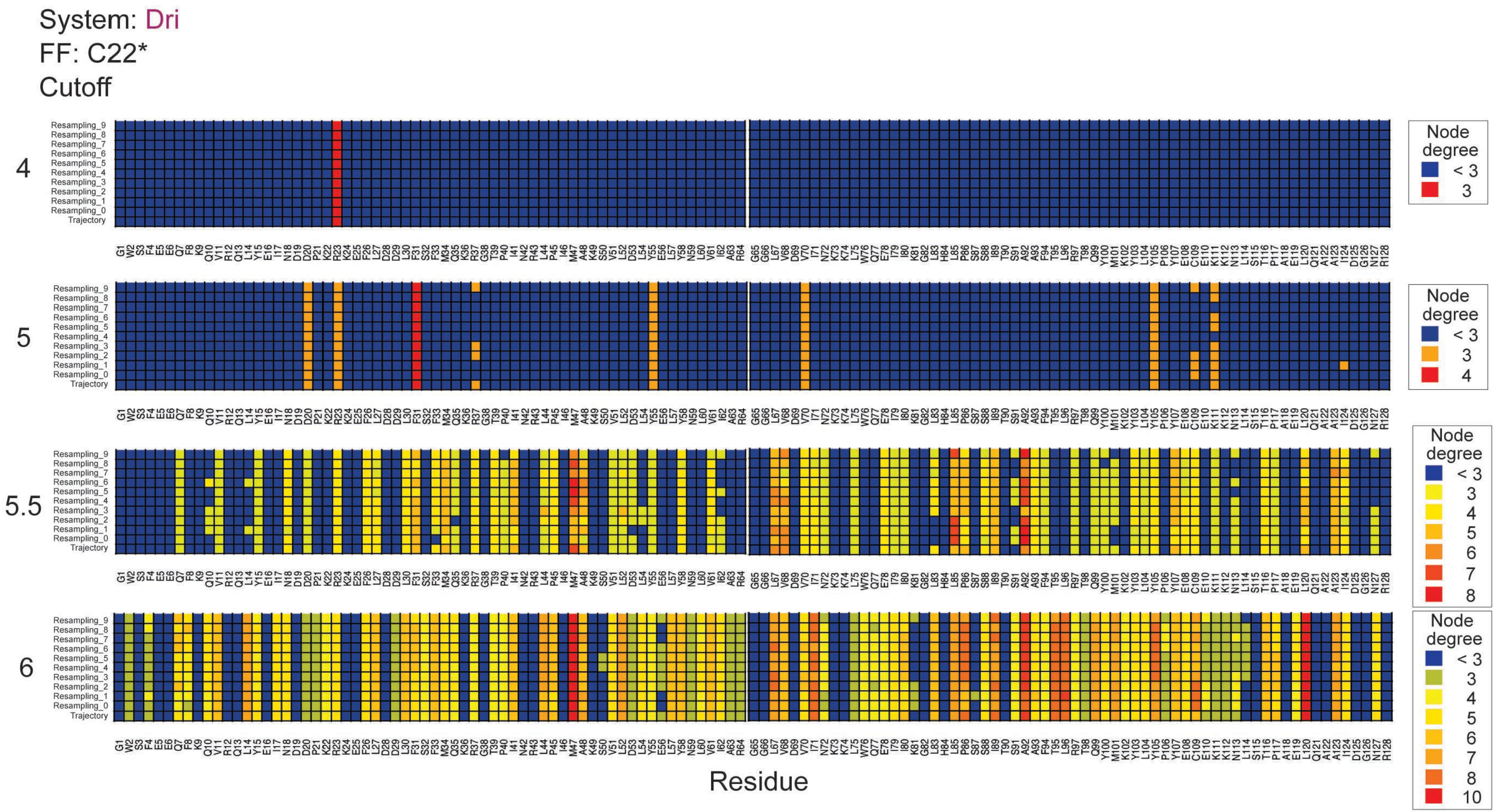
System: Dri
FF: C22*
Cutoff
4
5
5.5
6
Node degree
< 3
3
4
5
6
7
8
10
Trajectory
Residue

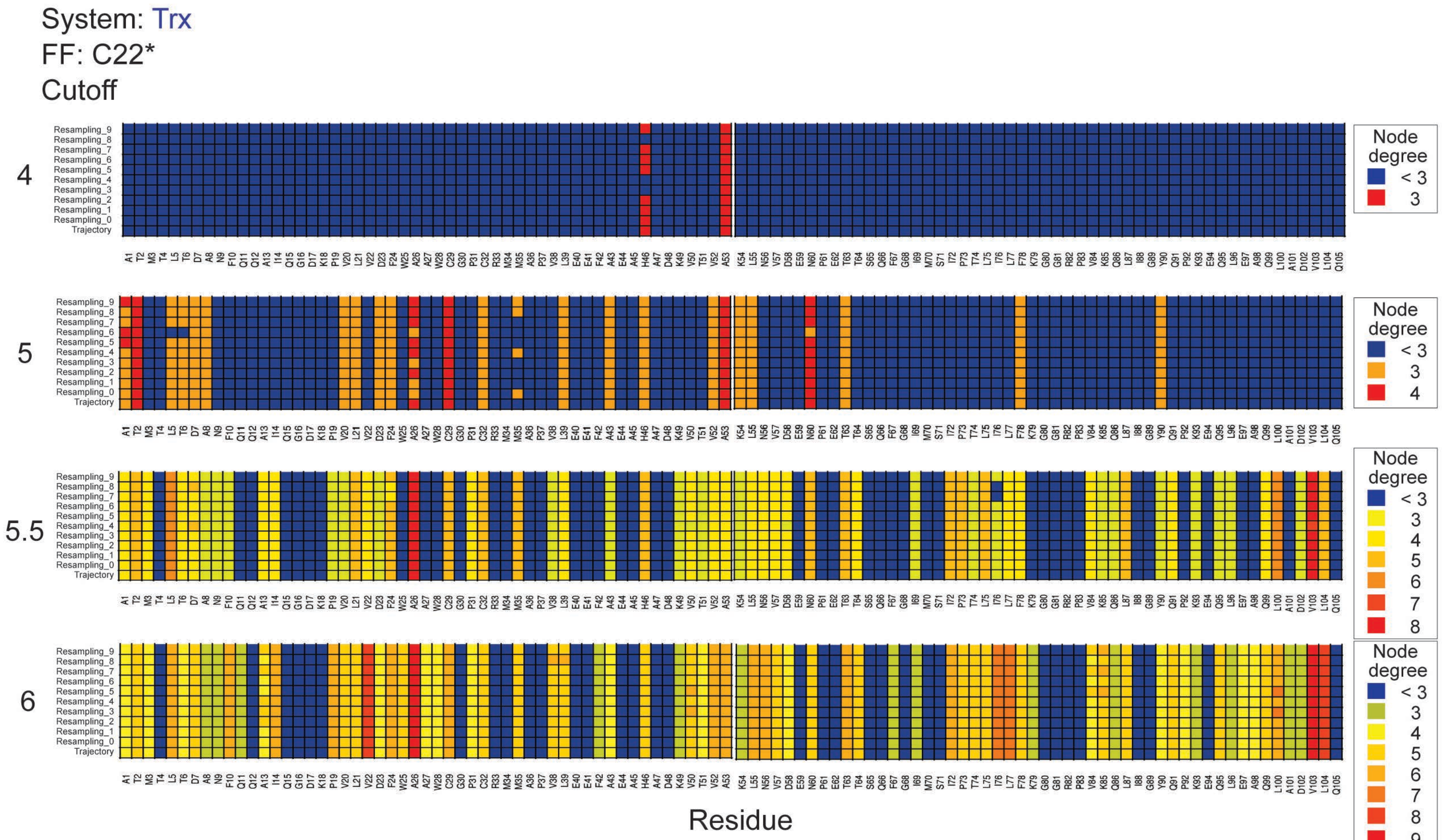

System: Trx
FF: C22*
Cutoff
4
5
5.5
6
Resampling_9
Resampling_8
Resampling_7
Resampling_6
Resampling_5
Resampling_4
Resampling_3
Resampling_2
Resampling_1
Resampling_0
Trajectory
Node degree
< 3
3
4
5
6
7
8
9
Residue

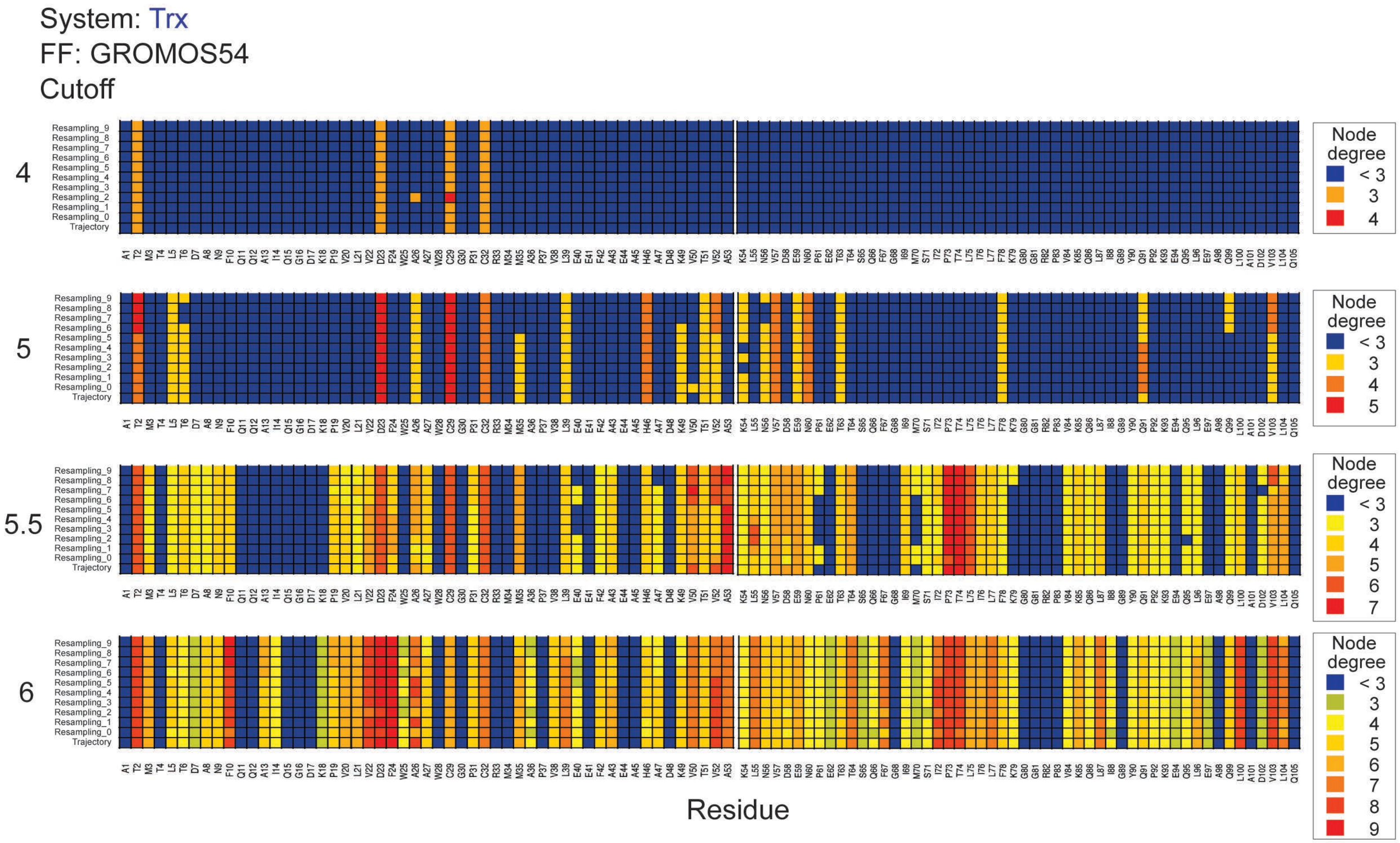
System: Trx
FF: GROMOS54
Cutoff
4
5
5.5
6
Node degree
Resampling_9
Resampling_8
Resampling_7
Resampling_6
Resampling_5
Resampling_4
Resampling_3
Resampling_2
Resampling_1
Resampling_0
Trajectory
Residue

Figure S3. **Heatmaps of connected components from all the MD simulations**. The residues belonging to each of the five most populated clusters as derived by each simulation using the Jackknife procedure are shown in the heatmaps.

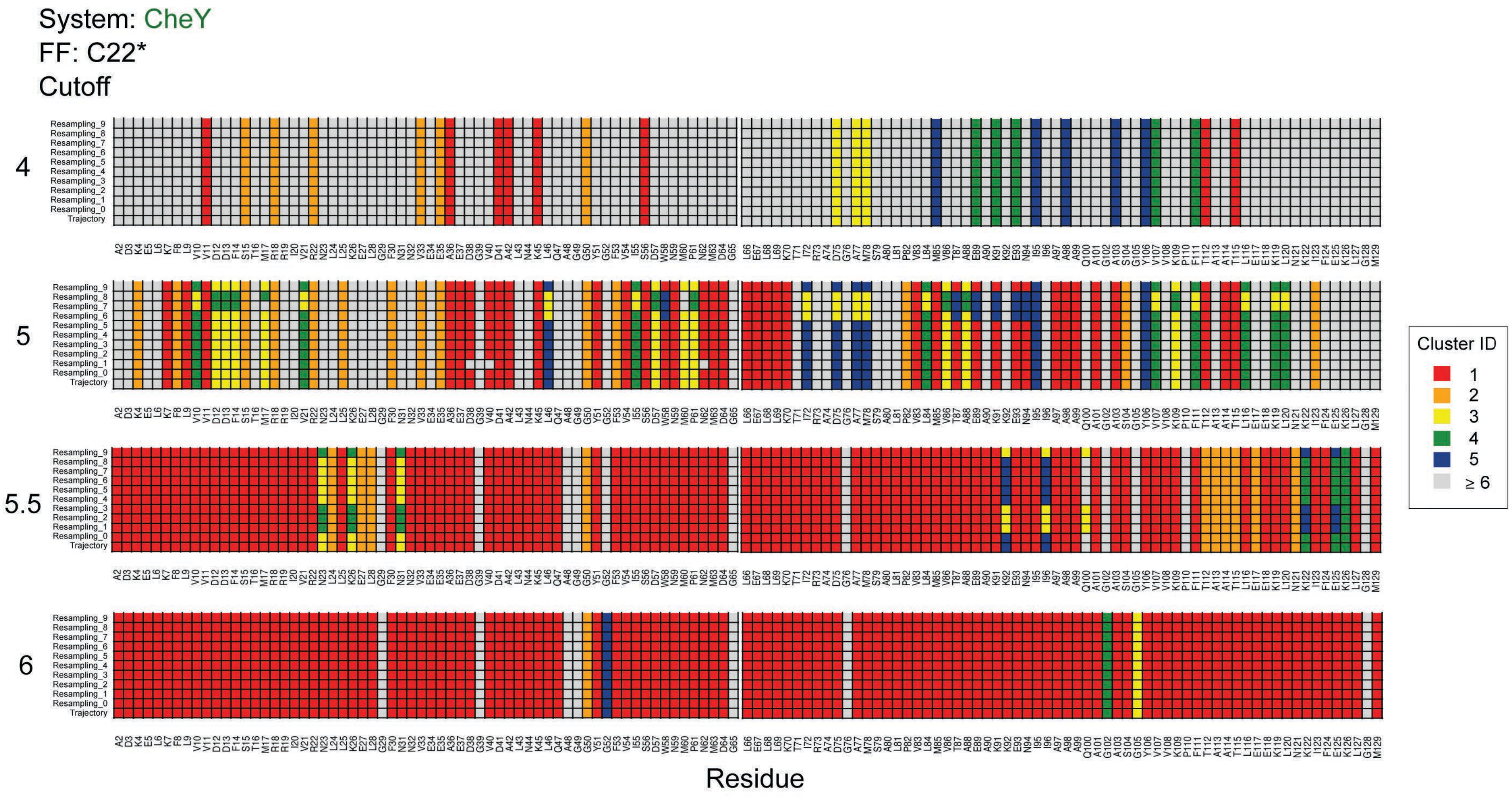

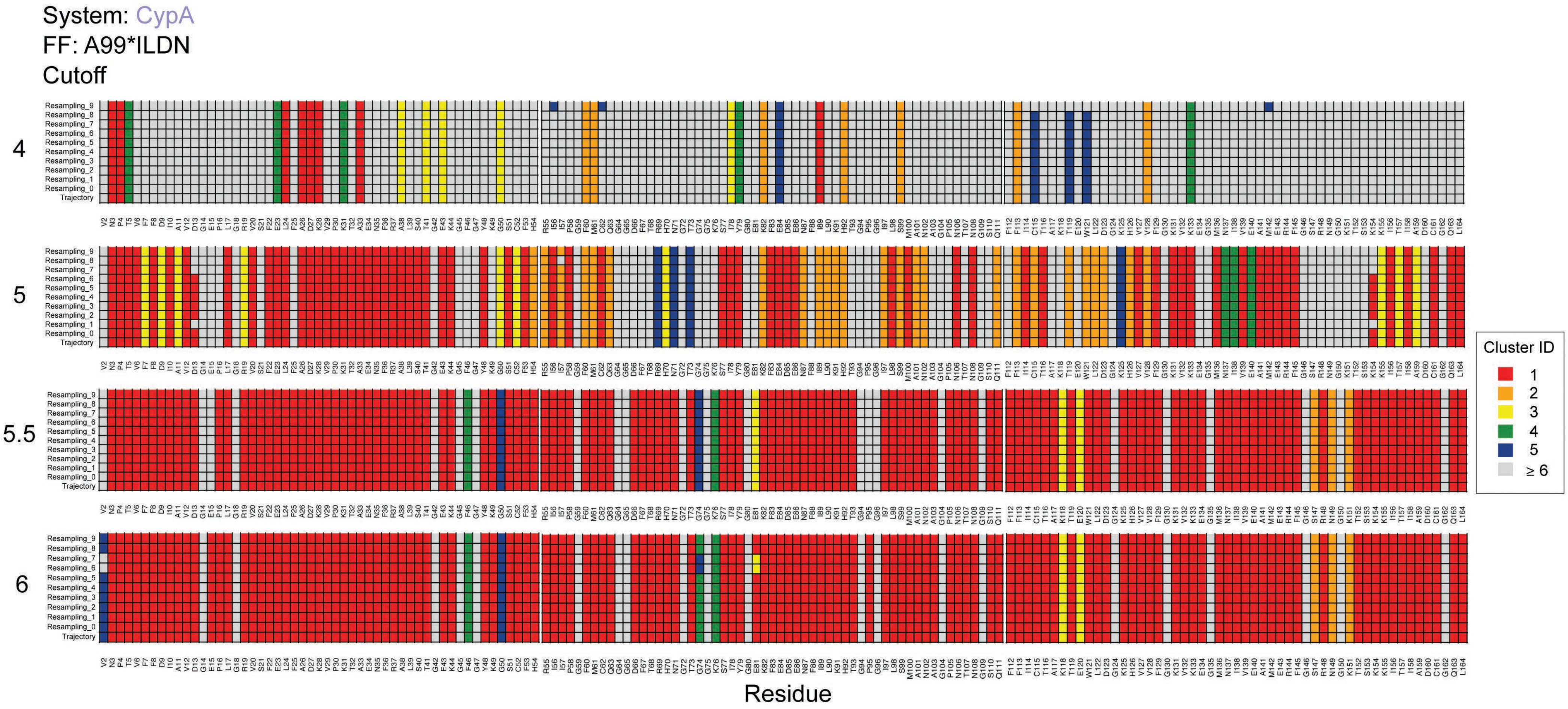
System: CypA
FF: A99*ILDN
Cutoff
4
5
5.5
6
Resampling_9
Resampling_8
Resampling_7
Resampling_6
Resampling_5
Resampling_4
Resampling_3
Resampling_2
Resampling_1
Resampling_0
Trajectory
Residue
Cluster ID
1
2
3
4
5
≥ 6

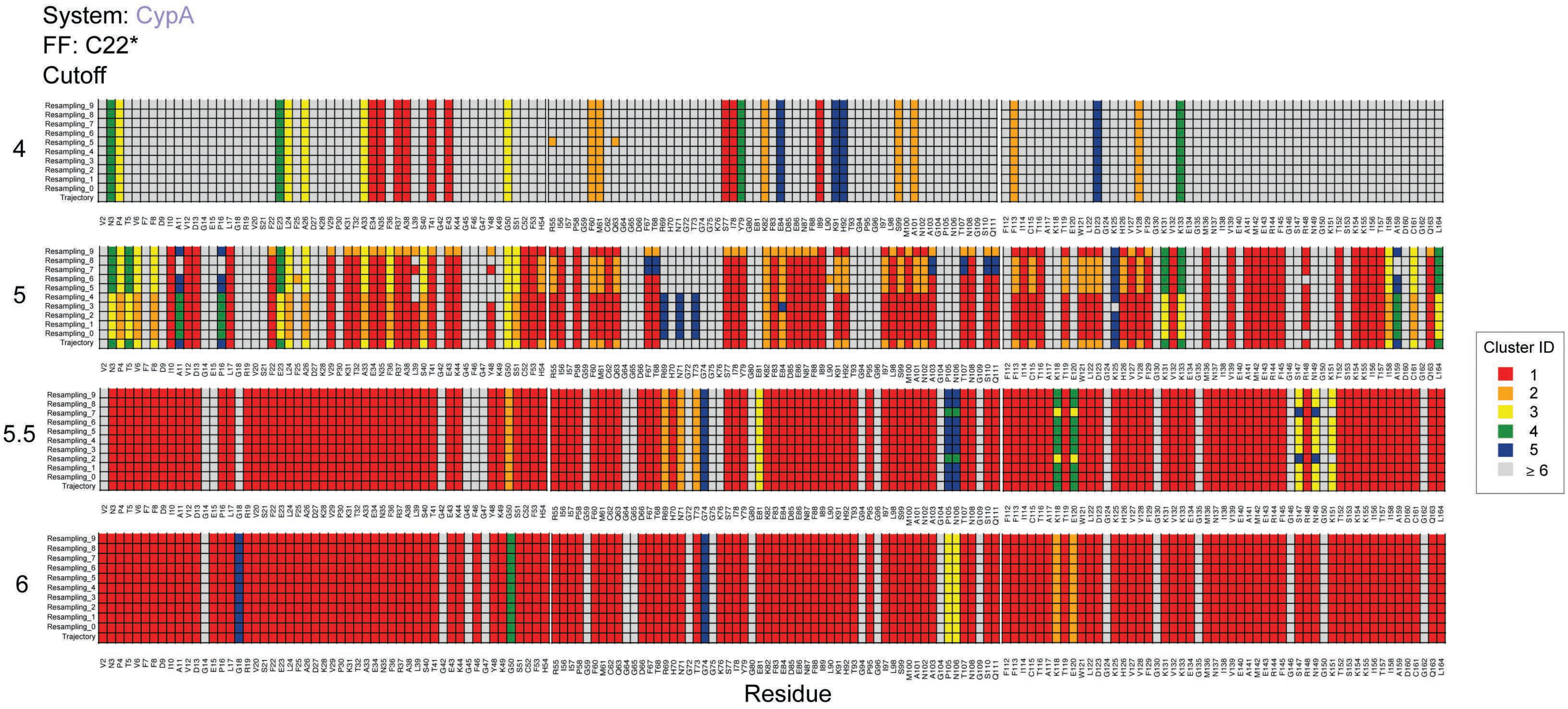
System: CypA
FF: C22*
Cutoff
4
5
5.5
6
Resampling_9
Resampling_8
Resampling_7
Resampling_6
Resampling_5
Resampling_4
Resampling_3
Resampling_2
Resampling_1
Resampling_0
Trajectory
Residue
Cluster ID
1
2
3
4
5
≥ 6

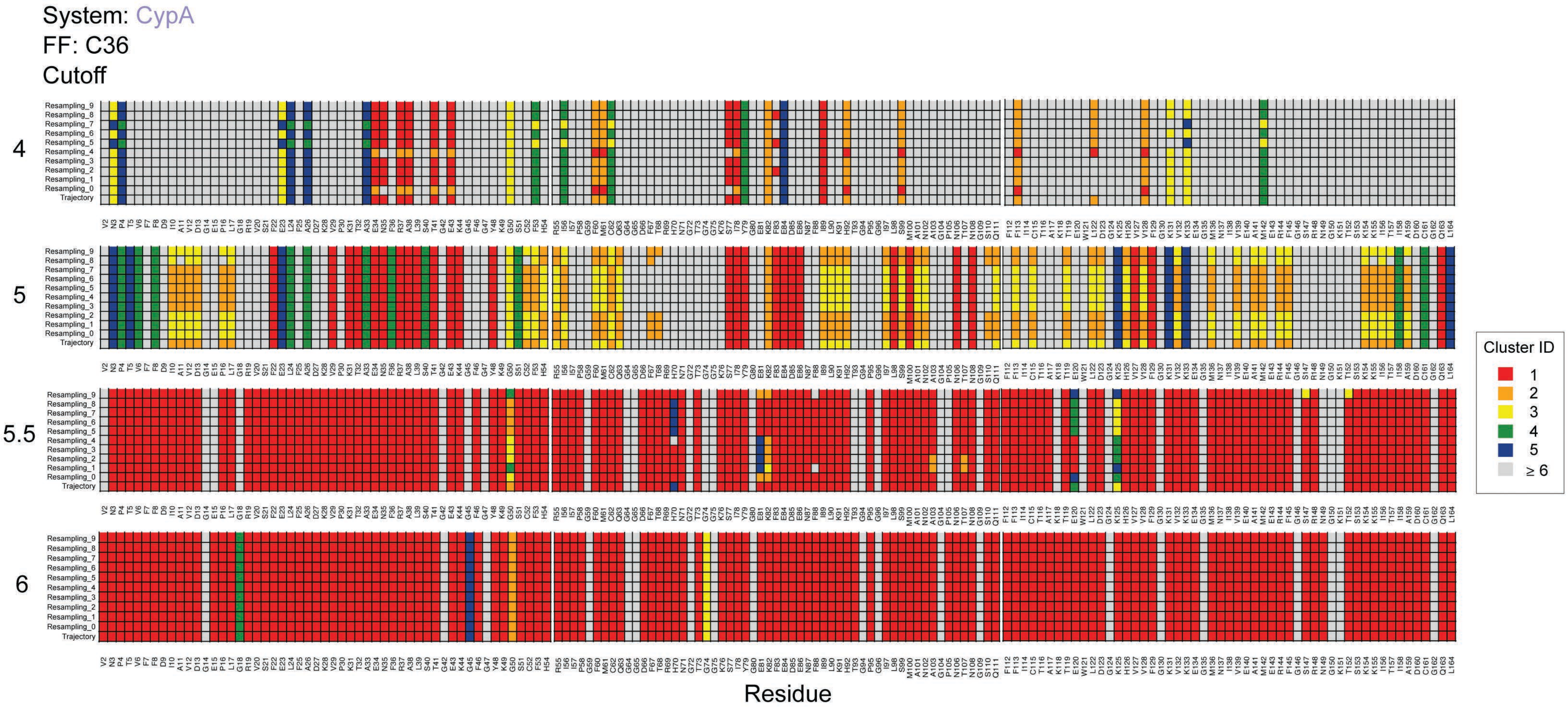
System: CypA
FF: C36
Cutoff
4
5
5.5
6
Resampling_9
Resampling_8
Resampling_7
Resampling_6
Resampling_5
Resampling_4
Resampling_3
Resampling_2
Resampling_1
Resampling_0
Trajectory
Residue
Cluster ID
1
2
3
4
5
≥ 6

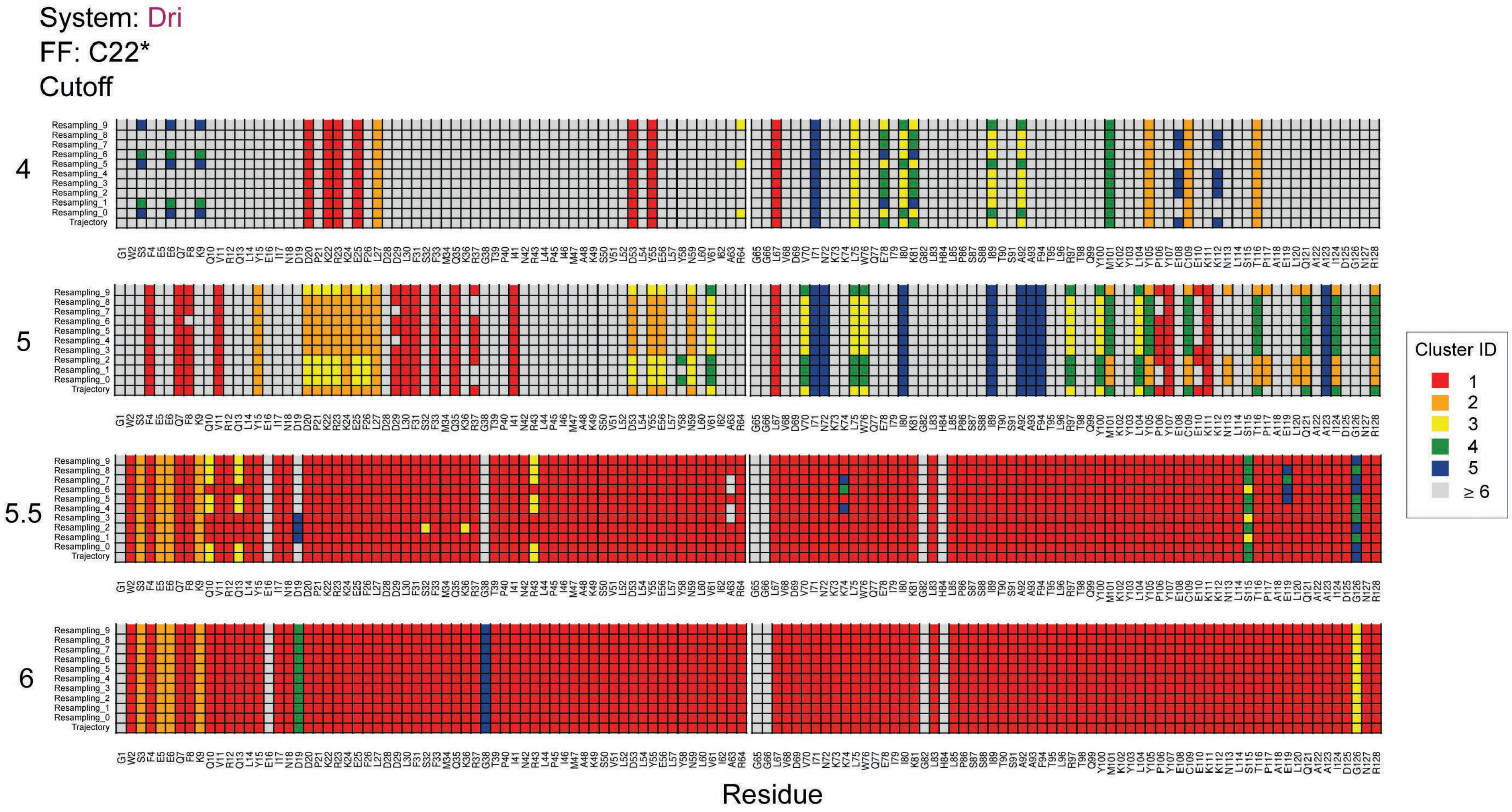

System: Dri
FF: C22*
Cutoff
4
5
5.5
6
Cluster ID
1
2
3
4
5
≥ 6
Residue

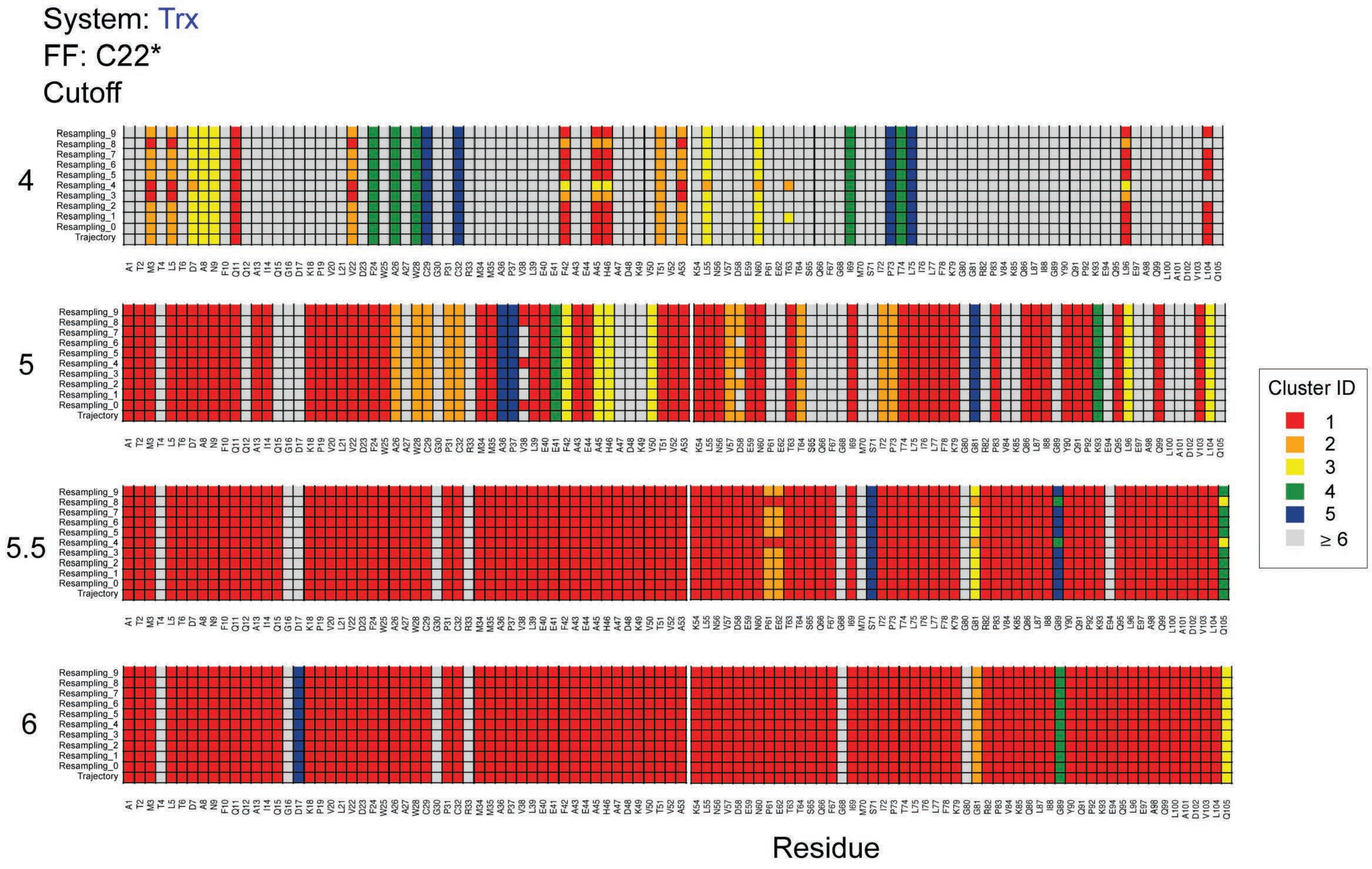

System: Trx
FF: C22*
Cutoff
4
5
5.5
6
Resampling_9
Resampling_8
Resampling_7
Resampling_6
Resampling_5
Resampling_4
Resampling_3
Resampling_2
Resampling_1
Resampling_0
Trajectory
Cluster ID
1
2
3
4
5
≥ 6
Residue

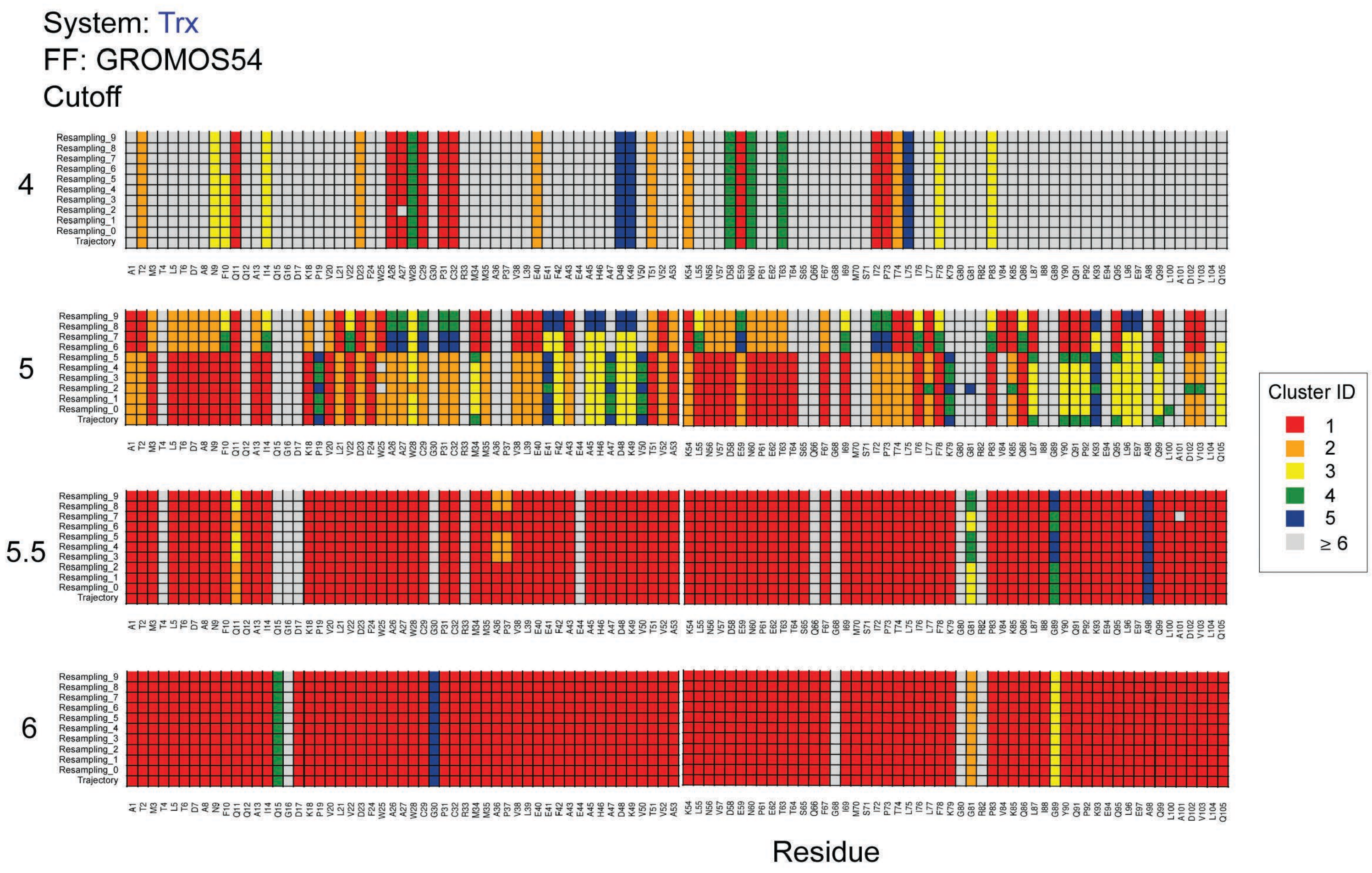

System: Trx
FF: GROMOS54
Cutoff
4
5
5.5
6
Resampling_9
Resampling_8
Resampling_7
Resampling_6
Resampling_5
Resampling_4
Resampling_3
Resampling_2
Resampling_1
Resampling_0
Trajectory
Cluster ID
1
2
3
4
5
≥ 6
Residue
